# Supplementary material for: Cell atlases and the developmental foundations of the phenotype
Source: PLoS Comput Biol. 2026 Feb 9;22(2):e1013944. doi: 10.1371/journal.pcbi.1013944 (PMC12904592; doi:10.1371/journal.pcbi.1013944)
Supplement: S1 Text — (PDF) [file pcbi.1013944.s001.pdf]

Supplement

Cell atlases and the Developmental Foundations of the Phenotype

Alicia Lou[1,2], Mónica Chagoyen[2¤b], Juan F Poyatos[1¤a*]

1 Logic of Genomic Systems Lab (CNB-CSIC), Madrid 28049, Spain

2 Computational Systems Biology group (CNB-CSIC), Madrid 28049, Spain

¤a Current Address: National Museum of Natural Sciences (MNCN-CSIC), Madrid 28006, Spain

¤b Current Address: Centro de Neurociencias Cajal (CNC-CSIC), Alcalá de Henares 28805, Spain

* Corresponding author: Juan F Poyatos (juanfpoyatos@csic.es)

Schematic overview of questions and metrics

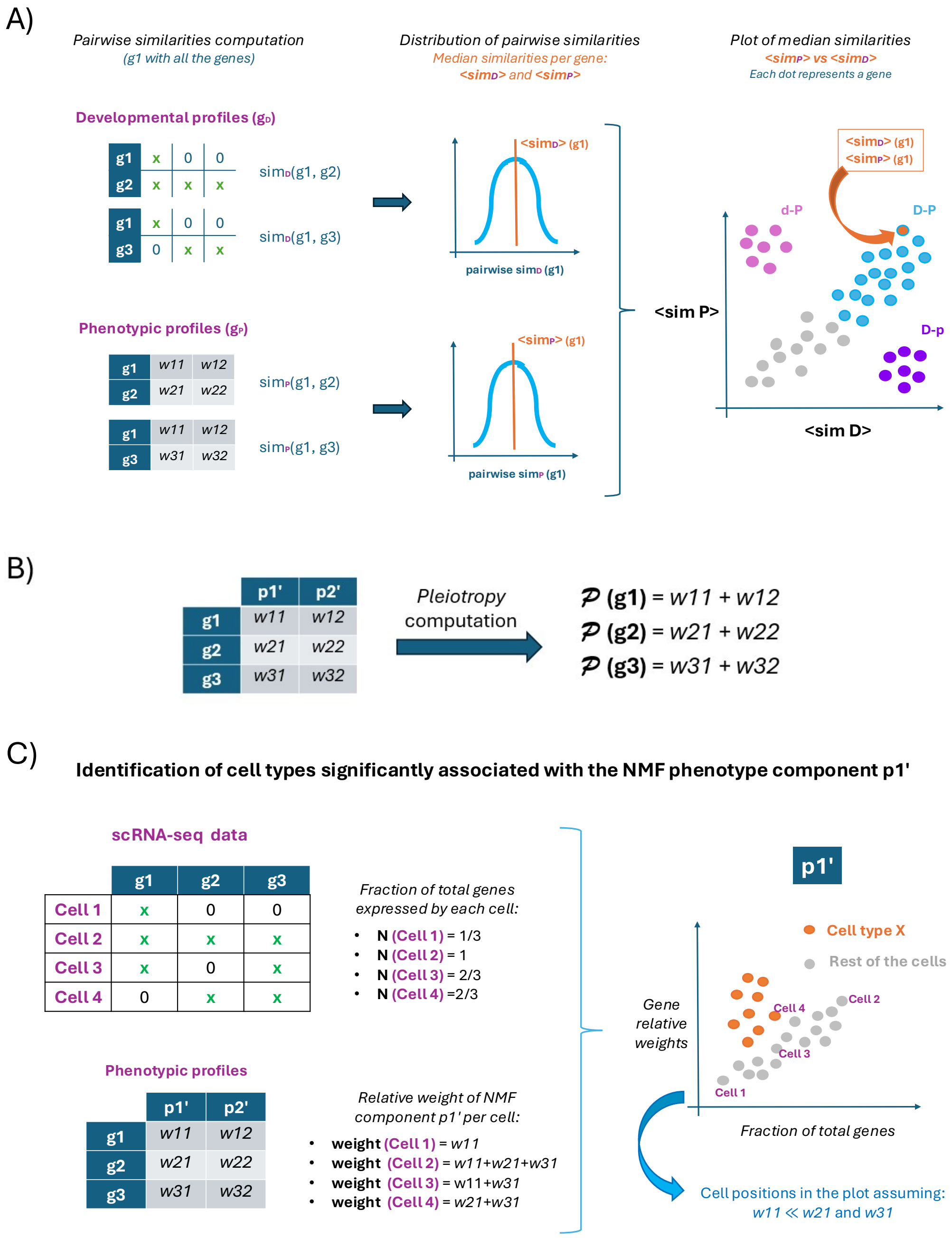

Fig A. Schematic representation of the metrics and their computation for each analysis section. A total of 8,233 genes were analyzed, representing 80% of the phenotype-associated genes listed in WormBase. The remaining 20% were not present in the developmental dataset from Packer *et al.* [1]. A) Computation of average phenotypic and developmental similarity per gene. Based on the construction shown in Fig 1A of the main text (where we generated the developmental gene expression matrix and the gene-phenotype association matrix via NMF), we computed the pairwise similarity of each gene to all others in both phenotype and developmental spaces: $\langle sim_{\mathrm{P}} \rangle$ and $\langle sim_{\mathrm{D}} \rangle$. We then plotted the distribution of these similarities and computed their median, which reflects how globally similar each gene is to others in each respective space. Finally, we plotted phenotypic versus developmental similarity per gene, revealing the $\mathcal{D}$–$\mathcal{P}$ rule trend and its deviations. B) Pleiotropy estimation. We computed gene pleiotropy as the sum of weights linking each gene to the phenotypic components derived from NMF. C) Example of phenotype-to-cell type association for a given NMF phenotype component: $p1'$. For each individual cell, we calculated the fraction of all genes it expresses and the relative contribution of phenotype component $p1'$ in that cell, based on the weights associated with $p1'$ and the set of expressed genes. Plotting gene relative weight versus the fraction of total genes per cell revealed which cells are most specifically linked to $p1'$. For instance, if weight $w11$ is low, but $w21$ and $w31$ are high, then cell 4—which expresses only two genes, both with strong association to $p1'$—would be considered tightly linked to that phenotype.

Extended analysis of phenotypic and developmental spaces

Phenotypic space

The Worm Phenotype Ontology (WPO) is an ontology of nematode phenotypes, representing relationships between a controlled vocabulary of phenotypic terms [2]. Terms are organized as a directed acyclic graph, where a term can be linked to several parental phenotypes. Fig B shows the ancestor terms of the phenotype 'G1 checkpoint variant'.

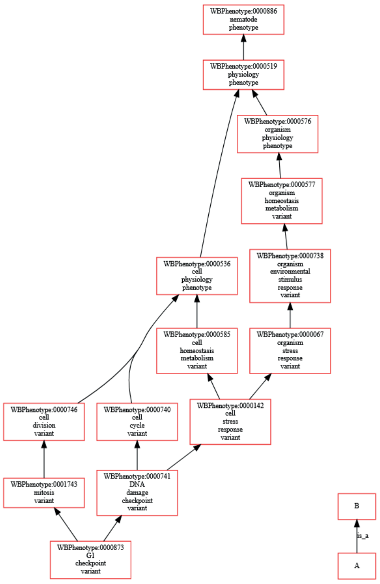

Fig B. Representation of the phenotypic space as a directed acyclic graph. This example illustrates the ancestors of the 'G1 checkpoint variant' (a specific term with no children), up to 'nematode phenotype' (the root term in the ontology).

Gene-phenotype associations

We downloaded gene-phenotype associations from the WormBase (`http://www.wormbase.org/`, version WS290; 10,407 genes linked to phenotypes and 119,901 associations). Most genes have a small number of phenotype annotations (Fig CA). All the associations correspond to genetic perturbations: 74% of them correspond to RNA interference (RNAi) studies and the rest (26%) to specific mutations (genetic allele variations) (Fig CB).

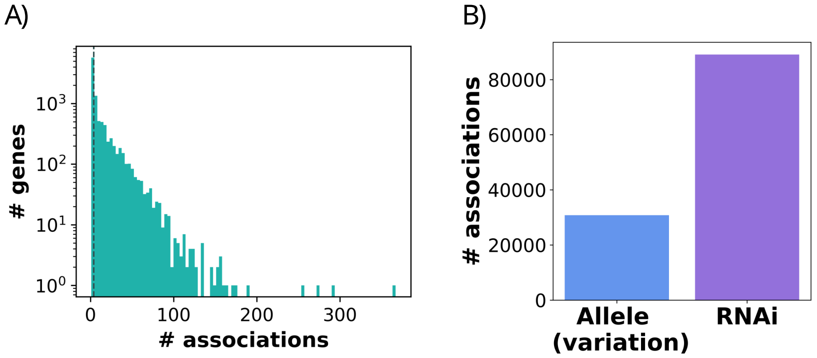

Fig C. Gene-phenotype associations. A) Distribution of the phenotype associations per gene. The dashed line indicates the median. B) Distribution of allele variation and RNA interference (RNAi) perturbations linked to phenotype associations.

Gene profiles in the phenotypic space

To map genes onto the multidimensional space of phenotypes, we first constructed a matrix of associations between genes and phenotypes, denoted as V (genes $\times$ phenotypes, Fig DA). When a gene is annotated with a phenotype in WormBase, we set the corresponding coordinate in V to 1, as well as the coordinates for all its ancestor terms in the ontology.

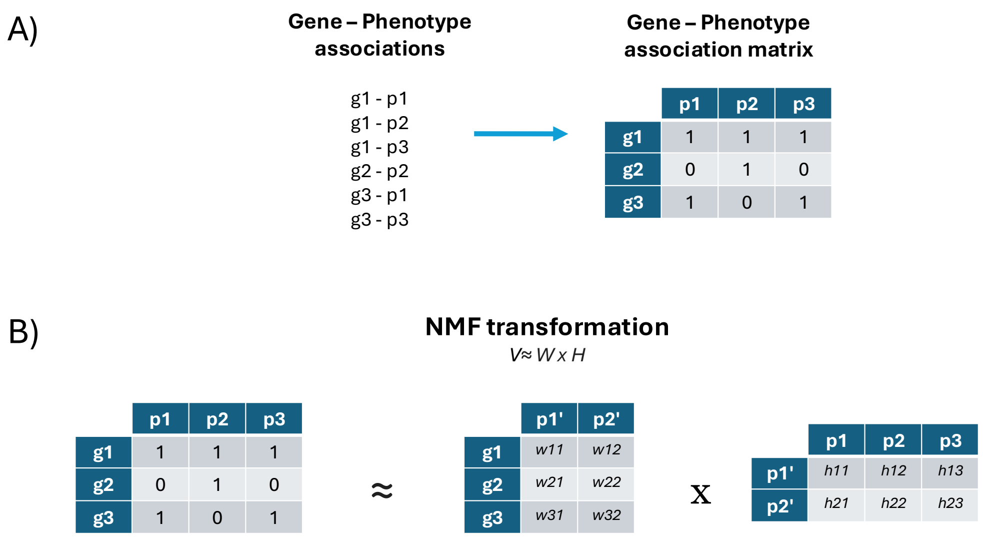

Fig D. Gene phenotypic profiles. A) Using gene-phenotype association data, we construct the gene-phenotype association matrix (V), which represents genes within the complete phenotypic space. B) Applying non-negative matrix factorization (NMF) to the gene-phenotype association matrix (V) produces two matrices: W and H.

Non Negative Matrix Factorization (NMF)

To reduce the dimensionality of the phenotypic space, while also reducing the redundancy of the phenotypes, we decomposed the V matrix (genes x phenotypes) by means of non-negative matrix factorization (NMF, [3]), where $V \approx W \times H$ (Fig DB). W represents genes in the reduced phenotypic space, and H captures the relationships between the new phenotypic components and the original phenotypes.

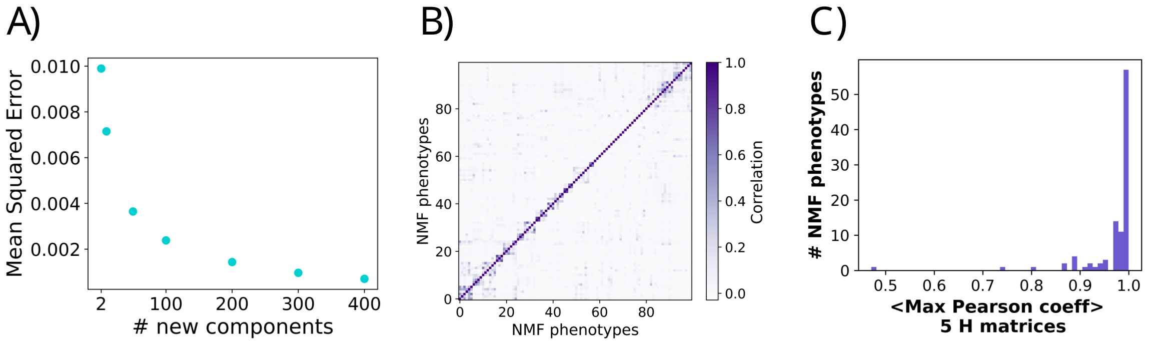

Fig E. NMF decomposition. A) Mean squared error with respect to the number of new phenotypic components. B) Correlation between the new components (H matrix). C) Distribution of NMF component stability. The figure shows the stability of NMF components, measured by comparing the reference H matrix to five new H matrices generated by rerunning the NMF algorithm (NMF involves an optimization, and this process is typically initialized with random values). For each component in the reference H, the highest Pearson correlation with components in the new H matrices was identified and averaged across the five runs. The resulting stability values are shown as a distribution.

To choose the number of new phenotypic components in the reduced space, we calculated the mean squared error of the reconstructed $W \times H$ matrix with respect to the initial phenotype matrix V (Fig EA; we selected 100 new components based on the curve elbow). Moreover, to check the independence of the new components we computed the correlation between them using the H matrix. The correlation between components is low (Fig EB). Fig EC quantifies their stability.

Developmental space

We built the development space from the single cell RNA sequencing data from Packer *et al.* [1]. Specifically, we downloaded the unique molecular identifier (UMI) count matrix, where rows represent individual cells and columns correspond to genes (89,701 cells and 20,222 genes). Fig FA shows the distribution of the total UMI counts per cell. The maximum UMIs in a cell is 126184, and the minimum is 484. The median = 1,557 UMIs. Fig FB shows the distribution of expressed genes per cell. The maximum number of cells expressing a gene is 82,912, and the minimum is 0. The median is 543 cells. To filter out empty droplets we discarded cells with <1000 UMIs. We also filtered out genes that were expressed in <3 cells. The shape of the filtered matrix is: 69,612 cells × 18,234 genes.

Each cell has an associated estimated embryo time. Most cells were annotated with a cell lineage, a cell type and/or a cell subtype by the authors (Fig FC). Cells with only lineage labels correspond to earlier embryo times (Fig FD). We relabeled each individual cell with our own 'cell type' label (229 distinct labels). To construct those labels we took all the possible combinations between the Packer *et al.* cell types and cell subtypes, including 'nan' labels for those cells that do not contain a cell type or cell subtype.

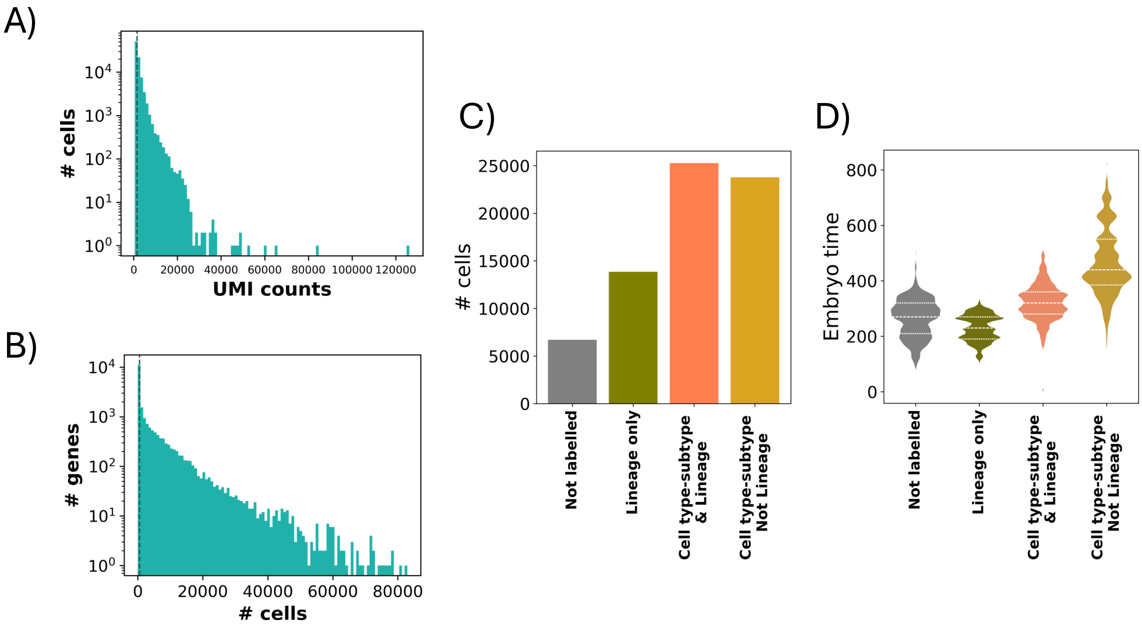

Fig F. UMI count matrix statistics. A) Distribution of total UMIs per individual cell. B) Distribution of total cells expressing a specific gene. In A) and B) dashed lines represent median values. C) Number of cells with different types of Packer *et al.* labels. D) Distribution of embryo times associated with those labels.

We included in our analysis only those cells with identified cell type or subtype information, result-

ing in a total of 49,049 cells. While the complete lineage of *C. elegans* is well characterized due to its relatively small and invariant number of cells, this level of detail is impractical to achieve in organisms with significantly larger and more variable cell populations. In such cases, the use of cell type or subtype labeling becomes essential for categorizing cellular identities and functions. This labeling not only facilitates comparative analyses within a given organism but also provides a framework for extrapolating findings across species, bridging the gap between well-characterized model organisms and more complex systems.

Definition of the developmental space

We first define a developmental space with two dimensions: embryo time and cell type. Each coordinate of this developmental space will be associated with a specific embryo time (t_i), with t_i up to 830 min after first cleavage, and one of 229 possible cell types. The dimension of this space is 136 time points $\times$ 229 cell types. Fig GA shows the number of cells in each developmental coordinate. Figs GB and GC show the number of cells in each embryo time and the number of cells in each cell type, respectively.

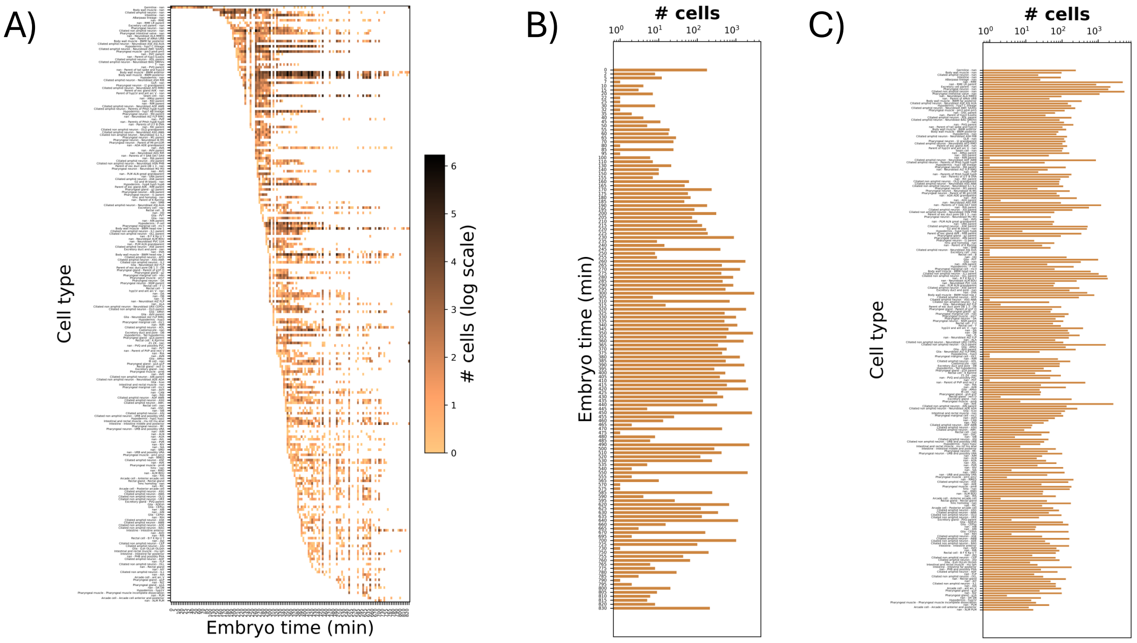

Fig G. Developmental space. A) Number of individual cells sampled at each developmental coordinate (embryo time $\times$ cell type). B) Number of cells sampled at each embryo time. C) Number of cells sampled for each cell type.

Gene expression profiles in the developmental space

To represent the expression profile of a gene, we reshaped this developmental space (2D) in a 1-D vector. Each developmental coordinate represents the fraction of cells with time i and cell type j, where the gene is expressed.

Potential information leakage between phenotypic and developmental space

To address the concern that our results could reflect implicit information sharing between the scRNA-seq data and phenotype ontology data, we explicitly evaluated whether gene–phenotype associations in our analysis could be biased by shared use of WormBase (WB) resources.

We first confirmed that the gene–phenotype associations used in our study were constructed independently of the scRNA-seq data. The Packer *et al.* scRNA-seq study [1] (reference WBPaper00057286), which underlies our developmental trajectory analysis, is not included in the WB phenotype association dataset (version WS290). Thus, the expression data we analyze were not used –directly or indirectly– to curate the gene–phenotype relationships in our matrix.

Moreover, the scRNA-seq study uses WB in three limited and well-defined ways: 1/technical processing, including read alignment to the WS260 reference transcriptome and quality control procedures (e.g., doublet detection), which are purely molecular and do not encode phenotypic knowledge, 2/cell-type and lineage annotation, based on previously established marker genes, and 3/nomenclature harmonization, involving re-annotation of earlier larval datasets to maintain consistent lineage labels across stages. Of these, only the use of marker genes for annotation presents a plausible route for subtle circularity.

To conservatively assess this risk, we focused on 55 phenotypes explicitly related to cell type and lineage –those most susceptible to marker-driven bias. Of the 8,233 genes in our gene–phenotype matrix, 281 were used as marker genes in the scRNA-seq annotations. Among these, only 68 genes are associated with the 55 lineage-related phenotypes. At the level of gene–phenotype associations –the quantity that directly enters our analysis– only 3,060 of 320,571 total associations ($\approx 1\%$) involve marker genes linked to lineage phenotypes. To ensure that no indirect effects were missed, we further evaluated all associations involving the 281 marker genes across all phenotypes in the matrix. Even under this deliberately conservative definition, these account for only 10,678 associations ($\approx 3\%$ of the total).

Therefore, across both phenotype-restricted and global analyses, the fraction of associations potentially influenced by shared marker usage is very small. This makes it unlikely that our reported relationships arise from information leakage or circular annotation. Instead, the associations we observe are dominated by signal from gene–phenotype relationships that are independent of the scRNA-seq data and its annotation pipeline.

Pairwise similarities

We analyzed the shared genes between the developmental and phenotypic spaces (n = 8,233 genes). Each gene is represented with two vectors (or profiles), one corresponding the developmental space ($\vec{g}_{\mathrm{D}}$) and the other with the phenotypic space ($\vec{g}_{\mathrm{P}}$) (see also main text). We computed all the pairwise similarities between genes in the two spaces using the cosine similarity measure obtaining sim_{D} and sim_{P} (Methods, main text).

We then calculated the median similarity of each gene with all the rest of genes in both spaces $\langle sim_{\mathrm{D}} \rangle$ and $\langle sim_{\mathrm{P}} \rangle$ (Fig H; S2 Table). In Fig HB we find three repeated similarities: 0.12 (338 genes), 0.14 (287 genes) and 0.07 (258 genes) which correspond to three groups of genes with very similar NMF phenotypic

profiles. The first group is characterized by the 97th NMF component (lethal, organism development variant). The second one, by component 6 (embryonic development variant, embryonic lethal), and the third, by components 13 (dauer metabolism phenotype, dauer lifespan variant) and 59 (organismal phenotype, anatomical phenotype).

The average developmental similarity is associated with the number of non-zero developmental coordinates. High $\langle sim_{\mathrm{D}} \rangle$ corresponds to more ubiquitously expressed genes (Fig HC).

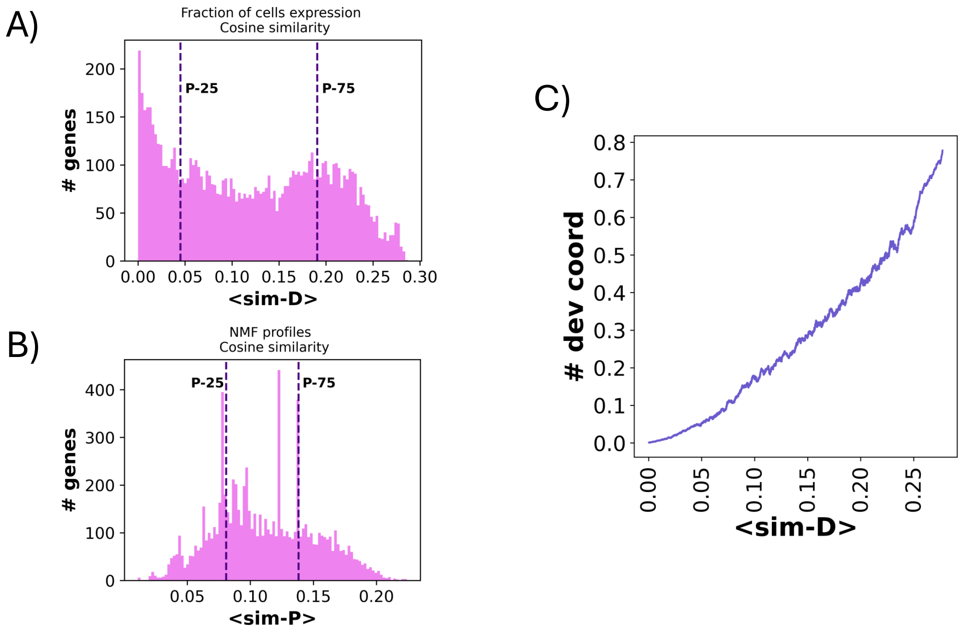

Fig H. Median similarities analysis. A) Distribution of $\langle sim_{\mathrm{D}} \rangle$. B) Distribution of $\langle sim_{\mathrm{P}} \rangle$. C) Number of non-zero developmental coordinates *vs.* $\langle sim_{\mathrm{D}} \rangle$.

$\mathcal{D}$–$\mathcal{P}$ rule and deviations using alternative representations

We also examined two binary representations of the developmental and phenotypic spaces as alternative representation. For the development space, we represented whether a gene was expressed in at least one cell at each developmental coordinate time i and cell type j. For the phenotypic space, we used the V matrix (see Section 1). We then computed the similarity of all pairs of genes in each binary space using the Jaccard similarity, Eq. (1).

$$sim_J(g_i, g_j) = \frac{g_i \cap g_j}{g_i \cup g_j}. \tag{1}$$

For each gene, we calculated the median similarity with all the rest of genes in both spaces $\langle sim_{\mathrm{D}}^{\mathrm{Jaccard}} \rangle$ and $\langle sim_{\mathrm{P}}^{\mathrm{Jaccard}} \rangle$ (distributions in Figs IA and IB). The $\mathcal{D}$–$\mathcal{P}$ rule holds true, regardless of how developmental and phenotypic expression is quantified. Figs IC and ID illustrate the $\mathcal{D}$–$\mathcal{P}$ rule using binary expression profiles from both developmental stages and phenotypic traits.

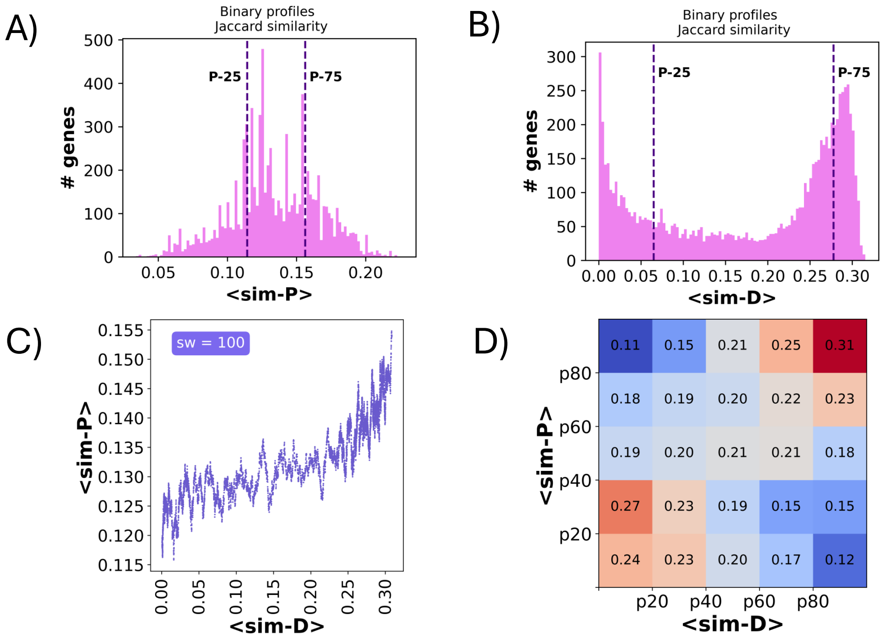

Fig I. Binary profiles analysis. We use Jaccard similarity. A) Distribution of $\langle sim_{\mathrm{D}}^{Jaccard} \rangle$. B) Distribution of $\langle sim_{\mathrm{P}}^{Jaccard} \rangle$. C) $\mathcal{D}$–$\mathcal{P}$ rule confirmation. D) Deviations of the $\mathcal{D}$–$\mathcal{P}$ rule.

Deviations of the $\mathcal{D}$–$\mathcal{P}$ rule

We calculated a Loess regression of $\langle sim_{\mathrm{D}} \rangle$ *vs.* $\langle sim_{\mathrm{P}} \rangle$ to study deviations from the $\mathcal{D}$–$\mathcal{P}$ rule (Fig JA). We then computed the residuals (distance to the regression fit) for each gene. Based on these residuals, we categorized genes into three groups: `D-P`, `D-p`, and `d-P` genes.

- `D-P` genes (blue, n = 1,322) adhere to the $\mathcal{D}$–$\mathcal{P}$ rule, with $\langle sim_{\mathrm{D}} \rangle$ values higher than the 75th percentile of the $\langle sim_{\mathrm{D}} \rangle$ distribution and residuals that do not deviate by more than one standard deviation (σ) from the mean of the residual distribution.

- `D-p` genes (purple, n = 168) exhibit low average phenotypic similarity, characterized by residual values below the 10th percentile of the residual distribution and $\langle sim_{\mathrm{D}} \rangle$ values higher than the 75th percentile of its corresponding distribution.

- `d-P` genes (pink, n = 245) show low developmental similarity, characterized by $\langle sim_{\mathrm{D}} \rangle$ values below the 25th percentile of the $\langle sim_{\mathrm{D}} \rangle$ distribution and residuals exceeding the 90th percentile of the residual distribution, indicating high phenotypic similarity.

In Fig JB, we plot the distributions of the non-zero developmental coordinates: `d-P` genes are expressed in few developmental coordinates (specifically expressed genes), while `D-p` and `D-P` genes are expressed in a high number of developmental coordinates (ubiquitously expressed genes).

We derived the *typical* developmental profile and phenotypic profile for each group of genes (`D-P`, `D-p` and `d-P`) (Fig 2, main text). For the (typical) developmental profile, we first calculated the fraction of cell types expressing a given gene within each embryo time. We then averaged (mean value) these expression profiles considering the set of genes of a given class (`D-P`, etc.). To obtain the (typical) phenotypic profile, we averaged the NMF phenotypic profiles of the genes within each group.

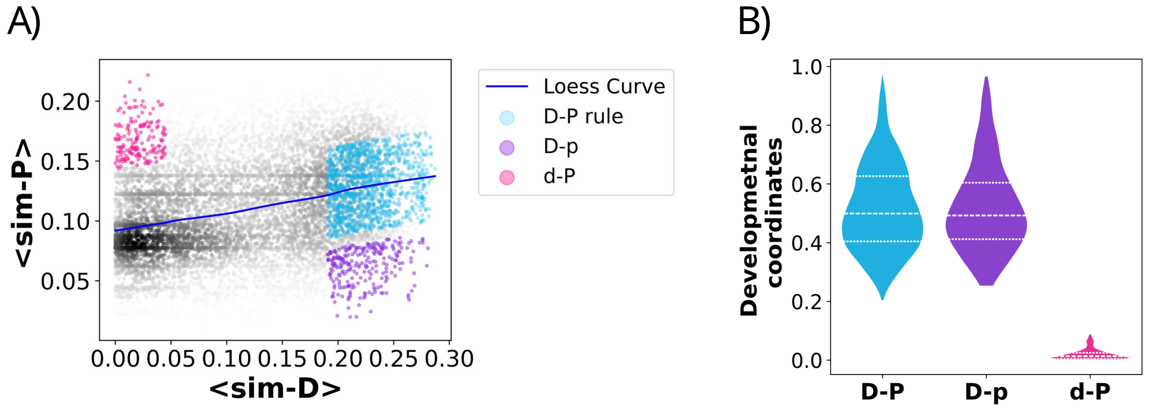

Fig J. **D-P rule deviations.** A) $\langle sim_{\mathrm{D}} \rangle$ *vs.* $\langle sim_{\mathrm{P}} \rangle$ per gene. The blue line is the Loess curve. We define three sets of genes to analyze: D-P genes (blue), D-p genes (purple) and d-P genes (pink). B) Distributions of non-zero developmental coordinates.

To illustrate deviations from the D-P rule, in Fig K, we compared the profiles of D-p and d-P (y-axis) against the D-P profile (x-axis). These comparisons are based on the typical profiles shown in Fig 2, main text. Specifically, each point in the scatter plots represents the values from these typical profiles. The D-P profile is plotted against itself as a reference line, serving as a baseline to identify quantitative differences. Points above or below this reference line indicate deviations: points above the line show where the values of D-p or d-P exceed the corresponding values of the D-P profile, while points below the line highlight where the values fall below the reference. These comparisons are shown in both developmental (Figs KA and KB) and phenotypic (Figs KC and KD) contexts, helping to reveal the extent to which the profiles of D-p and d-P deviate from the expected D-P pattern.

As expected, D-p and D-P genes show comparable expression levels at the same embryo times (Pearson's $r = 0.996$, p-value $= 3.66 \times 10^{-141}$, Fig KA). In contrast, d-P genes are only moderately correlated with D-P genes ($r = 0.50$, p-value $= 6.81 \times 10^{-10}$, Fig KB). This is due to the fact that d-P are expressed in a notably smaller number of developmental coordinates (Fig JB). Some of them are generally expressed at very low levels, while others are typically lowly expressed but show high or moderate expression in specific coordinates.

For the phenotypic profiles, the correlation between d-P and D-P genes ($r = 0.76$, p-value $= 5.20 \times 10^{-20}$, Fig KC) is high, although lower than the conservation of developmental context (D-P *vs.* D-p). This is explained by the fact that D-P and D-p genes show $\langle sim_{\mathrm{D}} \rangle$ within the same range (Fig JA), while $\langle sim_{\mathrm{P}} \rangle$ of d-P genes is, on average, higher than that observed in the D-P genes. The phenotypic correlation between D-p and D-P genes is moderate ($r = 0.40$, p-value $= 2.69 \times 10^{-5}$).

To better understand the differences between these two groups in the phenotypic space, we selected the phenotypic components with a higher value in the D-p profile than in the D-P one (components #96, #72 and #86; Fig 2, main text). These components are associated with 'neuron development variant' (comp. #96), 'neuron morphology variant' (comp. #72) and 'cytoplasmic appearance defective early embryo' and 'cell-cell contacts abnormal' (comp. #86). Although D-p genes are expressed broadly during development (Fig JB), their impact in the phenotype is highly specific, explaining their lower $\langle sim_{\mathrm{P}} \rangle$

values.

Component #87 (associated with 'germ cell cytoplasmic morphology variant') is characteristic of both `D-P` and `D-p` groups but not of the `d-P` group. This is consistent with the observation that `D-P` and `D-p` genes are highly expressed during germline development, whereas `d-P` genes are not.

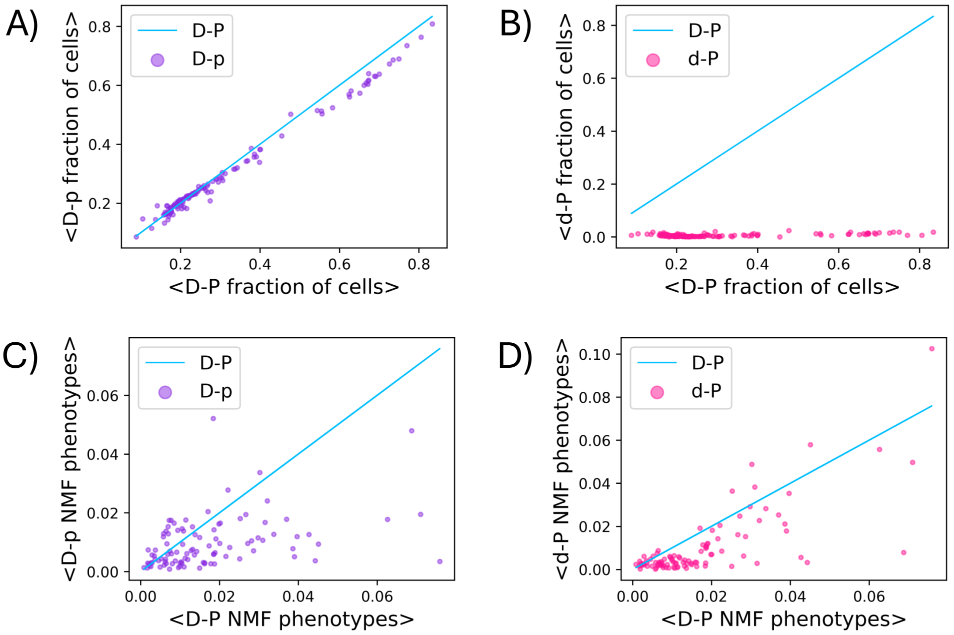

Fig K. Comparison of `D-P`, `D-p`, and `d-P` typical profiles in developmental and phenotypic contexts. In the developmental profile scatter plots (A and B), each point represents the average fraction of cell types expressing genes at each embryonic stage, comparing the subsets `D-p` or `d-P` to `D-P` discussed in Fig 2 (main text). In the phenotypic context (C and D), each point in the scatter plots corresponds to the average value associated with each NMF phenotype in the profiles of the `D-p` or `d-P` subsets, compared to `D-P`. A) `D-p` *vs.* `D-P` developmental profile values. B) `d-P` *vs.* `D-P` developmental profile values. C) `D-p` *vs.* `D-P` phenotypic profile values. D) `d-P` *vs.* `D-P` phenotypic profile values. The blue line is the representation of the `D-P` profile against itself. Deviations in the average profiles of `D-p` and `d-P` compared to `D-P` are highlighted by points falling above or below this line. All panels show significant correlations between `D-P` profiles and their deviations. In panel B, the moderate correlation ($r = 0.50$, $p = 6.81 \times 10^{-10}$) is visually masked due to fewer cell types expressing genes in the `d-P` profile, which compresses the plot scale.

To complete the analysis, we performed a phenotype enrichment for each group of genes. The list of enriched phenotypes (p-value $< 1{\times}10^{-3}$) is provided in S4 Table. Enriched phenotypes in the `D-P` group and `d-P` groups include 'lethal', 'organism development variant', 'growth variant' and 'sterile'. The enriched phenotypes in the `d-P` and `D-P` classes are predominantly systemic and general, reflecting their organism-wide impact. The majority of enriched phenotypes in the `d-P` group are also enriched in the `D-P` group.

The `D-p` group is enriched in 'body wall muscle myosin organization defective', 'neuron migration variant' and 'neurite development variant' which generally refer to more specific phenotypes (muscle and neuron). Enriched phenotypes explain only a small percentage of genes within this group. No overlap is observed between the most enriched phenotypes (p-value $< 1 \times 10^{-4}$) of `D-p` and `D-P`.

Finally, Gene Ontology (GO) analysis revealed additional differences between the three groups. (S5 Table): **i**/`D-P` genes are enriched in a large number of GO terms, related to housekeeping functions: 'intracellular organelle', 'cytoplasm' and 'ribonucleoprotein complex' (in terms of cellular localization), 'biosynthetic process', 'RNA processing' and 'translation' (in terms of biological processes) and 'RNA binding' and 'structural constituent of ribosome' (in terms of molecular functions), **ii**/ `d-P` genes are

enriched in only a small number of GO terms, revealing a general heterogeneity of functions. The enriched terms refer to 'nucleosome' and 'chromatin', 'extracellular ligand-gated monoatomic ion channel activity' and 'collagen trimer'. Very specific cell functions that are needed for the viability of the organism, and **iii/** `D-p` genes are also enriched in a small number of GO terms, most related to cellular components: 'cytoplasm', 'endomembrane system', 'vacuole' and 'nucleolus'; and few biological processes 'rRNA processing' and 'protein ufmylation'. Cell components which are present in all the cells but might play specific roles in certain cell types.

Pleiotropy

The pleiotropic score $\mathbb{P}$ is calculated by adding the scores associated with each gene in the matrix W (obtained by NMF). We defined two classes of genes with extreme $\mathbb{P}$ values based on the pleiotropy distribution (Fig L): pleiotropic genes (n=412, above 95th percentile) and non-pleiotropic genes (n=418, below 5th percentile).

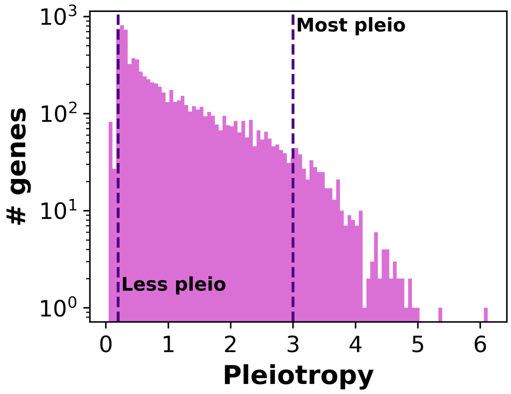

Fig L. Pleiotropy distribution. Dashed lines indicate the 5th and 95th percentiles that define non-pleiotropic and pleiotropic genes, respectively.

The biased induced by the hierarchical nature of the ontology

The most specific phenotypes (i.e., those without children) have different depths in the WPO hierarchy (distance from the root term, 'nematode phenotype', Fig MA), depending on the branch of the ontology. Thus, genes annotated with deeper terms in the hierarchy will generally be annotated with more phenotypes, leading to an apparent higher pleiotropy value. NMF partially eliminates this bias (Fig MB). We compared the pleiotropy ($\mathbb{P}$), based on NMF profiles, with respect to that of the original phenotypic space (defined as the sum of the corresponding row values in the V matrix). Genes associated with specific phenotypes with more than 25 ancestors are represented as orange dots (biased genes). Most of them fall below the linear regression line, indicating that the bias was partially eliminated by NMF.

As NMF is sensitive to initialization, we evaluated the stability of $\mathbb{P}$ across different runs (Fig MC). To quantify this, we calculated the coefficient of variation (CV=$\frac{\mu}{\sigma}$), where μ is the mean and σ is the standard deviation of the five $\mathbb{P}$ values for each gene. The CV values remained below 0.3, confirming the consistency in the $\mathbb{P}$ values to initialization variability. Finally, Fig MD shows the correlation between $\mathbb{P}$ and the number of non-zero components in W (Pearson's r =0.92, p-value=0.0).

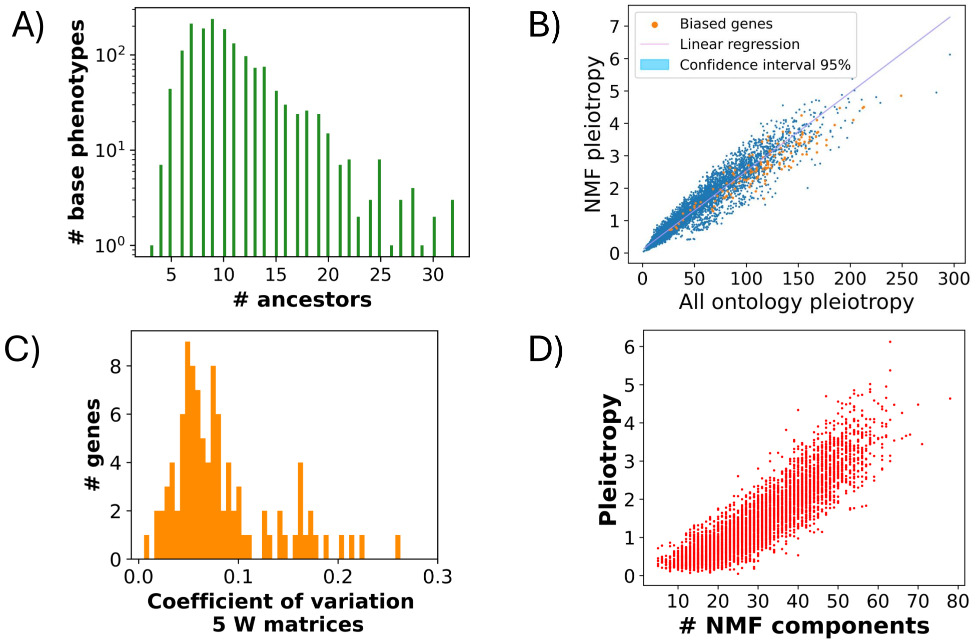

Fig M. Redundancy, pleiotropy bias, and pleiotropic score stability. A) Depth of most specific phenotypes in the WormBase Phenotype Ontology. B) NMF (W) pleiotropy vs. V pleiotropy. Orange dots represent those genes associated with at least one of the most specific phenotypes that have more than 25 ancestors in plot A. The major part of those genes fall below the linear regression. C) Coefficient of variation of pleiotropy $\mathbb{P}$ between five NMF runs. D) Pleiotropy $\mathbb{P}$ *vs.* number of non-zero NMF components (W matrix) per gene.

Pleiotropic and non-pleiotropic genes

In Fig NA, we present the distributions of non-zero developmental coordinates where pleiotropic and non-pleiotropic genes are expressed. Most non-pleiotropic genes are expressed in very few developmental coordinates. Figs NB and NC represent the fraction of cell types *vs.* embryo times where a gene is expressed. A gene is considered expressed in a cell type or time if at least one corresponding cell shows expression. This can overestimate gene expression, making genes with limited expression in specific developmental coordinates appear broadly expressed across many cell types or stages. Thus, some non-pleiotropic genes are also expressed in a high fraction of cell types and embryo times (Fig NC). Most pleiotropic genes are expressed in a high fraction of cell types and embryo times (Fig NB).

Next, we compared pleiotropic and non-pleiotropic genes in terms of their enrichment in phenotypes (S6 Table) and GO terms (S7 Table). We find 214 enriched phenotypes for pleiotropic genes. Those with the lowest p-value are: 'organ system development variant', 'cell phenotype' and 'organ system morphology variant'. Some more specific enriched phenotypes are: 'cell homeostasis metabolism variant', 'fertility reduced', 'gametogenesis variant', 'gonad morphology variant', 'vulva morphology variant', 'vesicle trafficking variant', 'feeding behavior variant' and 'organism segment morphology variant'. There is just one enriched phenotype for non-pleiotropic genes: 'organism metabolism processing variant'.

With respect to the GO terms, the pleiotropic genes are enriched in a high number of them including: 'intracellular anatomical structure', 'cellular process', 'anatomical structure development', 'cellular component organization or biogenesis' and 'positive regulation of biological process'. Non-pleiotropic genes are enriched in just 6 terms such as 'zinc ion binding' and 'DNA-binding transcription factor activity'. Those terms associate with a small subset of non-pleiotropic genes.

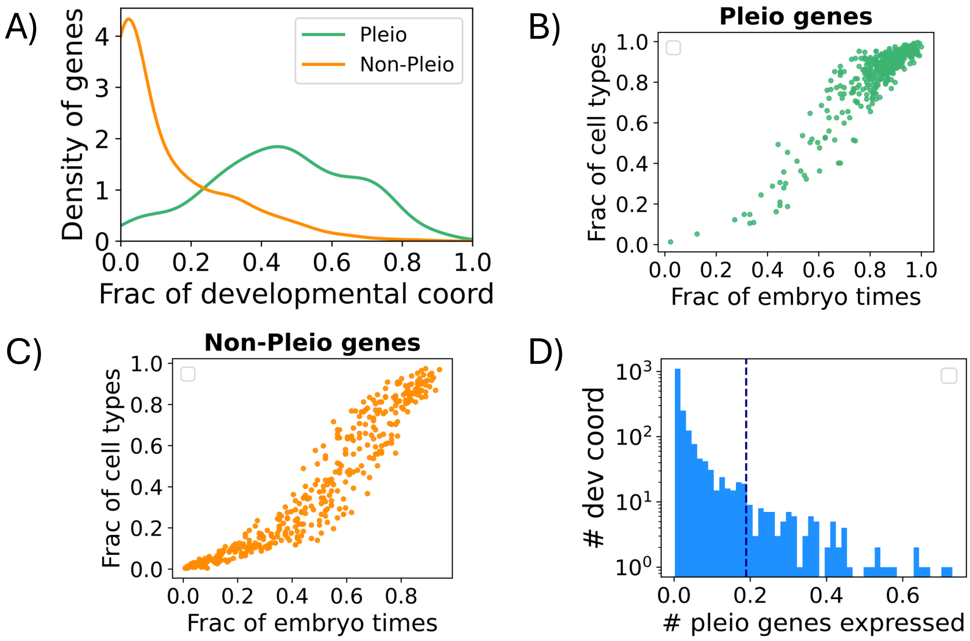

Fig N. Pleiotropic and non-pleiotropic genes comparison in development. A) Distributions of the fraction of non-zero developmental coordinates. B-C) Scatter plot of the fraction of non-zero cell types *vs.* the fraction of non-zero embryo times. B) Non-pleiotropic genes. C)Pleiotropic genes. D) Distribution of the fraction of pleiotropic genes significantly over-expressed in each developmental coordinate. Dashed line marks top 100 values.

Relationship between pleiotropy and development

To examine to what extent pleiotropic genes are particularly expressed at certain developmental coordinates, we first computed the probability of expression of a given gene g_a as the ratio between the total number of cells in which it is expressed, n_a, and the total number of sampled cells in the full developmental space, N, $p_a = \frac{n_a}{N}$.

Following this, the probability that a gene g_a is expressed in a specific coordinate (i, j) is given by the binomial distribution $P(X = x_{ij}) = \binom{m_{ij}}{x_{ij}} p_a^{x_{ij}} (1 - p_a)^{m_{ij} - x_{ij}}$. This is the probability of having x_{ij} cells expressing the gene in a coordinate (i, j) with a total of m_{ij} sampled cells. Given this distribution, we introduce the z-score$(g_a, i, j) = \frac{x_{ij} - m_{ij} p_a}{\sqrt{m_{ij} p_a (1 - p_a)}}$. We also calculated an over-expression p-value by adding the probability of obtaining a number of cells equal or higher than x_{ij} in a coordinate: p-value$(g_a, i, j) = P(X \geq x_{ij}) = 1 - P(X < x_{ij}) = 1 - P(X \leq x_{ij} - 1)$. To assess whether a gene is over-expressed at each coordinate, we used the criteria z-score>2 and p-value<0.001.

For each coordinate, we computed the fraction of pleiotropic genes over-expressed based on the previous criterion. Fig ND depicts the distribution of developmental coordinates with a given fraction of over-expressed pleiotropic genes. From this data, we focused on the top 100 coordinates with the highest values (above the dashed line in Fig ND) for further analysis (Section 3, main text, Fig 3C). The standout cell types in Fig 3C of the main text are:

- All the intestine cell types.
- Pharyngeal 1/12: ‘Pharyngeal marginal cell - mc1’.
- Body Wall Muscle 4/6: ‘Body wall muscle - nan’, ‘Body wall muscle - BWM anterior’, ‘Body wall

muscle - BWM posterior' and 'Body wall muscle - BWM head row 1'.

- Neuron: (3/140) 'Ciliated amphid neuron - nan', 'Ciliated non amphid neuron - nan', and 'Ciliated amphid neuron - Neuroblast ASE ASJ AUA (precursor)'.
- Excretory cells (1/11): 'Excretory cell - nan'.
- Hypodermis (2/11): 'Hypodermis - hyp7 C lineage' and 'Hypodermis - hyp7 AB lineage'
- Precursor cells: 'Germline - nan', 'Seam cell - nan' and 'M cell - nan'.

Note that 8 of the 20 highlighted cell types could not be annotated with cell subtype and appear with the label 'nan', which could be related to early cell types (partially differentiated, but not fully specific).

Additionally, we identified relationships between specific phenotypes enriched with pleiotropic genes and the highlighted cell types shown in Fig 3C of the main text. For example, 'cell homeostasis metabolism variant' can be associated with Excretory cells (removal of metabolic waste) and Intestine cells (digestion and absorption). Also, 'Hypodermis - hyp7 AB lineage' play an important role in vulva formation ('vulva morphology variant'), as they interact with vulva precursor cells. 'Fertility reduced' can be related to Germline. 'Feeding behavior variant' can be associated with the role of Ciliated Amphid Neurons (detecting food-related chemical cues), Intestinal Cells (Middle, Posterior, Anterior for food processing), Pharyngeal Muscles (pm3, pm4, pm5; crucial for food ingestion) and Body Wall Muscles (BWM Anterior, BWM Posterior; locomotion toward food). 'Organism segment morphology variant' can be related to Hypodermis (hyp7 AB lineage, hyp7 C lineage) and Body Wall Muscles (BWM anterior and posterior) important for segment organization, and Seam Cells, for the maintenance of segment boundaries.

With respect to the Fig 3D, main text, we observed that genes with higher $\langle sim_{\mathrm{P}} \rangle$ tend to have higher pleiotropy values (Fig OA). This explains how the `d-P` genes are (in general) more pleiotropic than the `D-p` ones (Fig OB). The distributions are significantly different (KS=0.21, p-value=9.36x10^{-5}).

Alternative measures of pleiotropy

We compared our measure of pleiotropy with some earlier work. Zou *et al.* [4] provided a list of 19 highly pleiotropic genes. We just found 6 genes from that list in our set of ≈8,000 analyzed genes. 4 of those genes were within *our* pleiotropic genes. Xiao *et al.* [5] analyzed a set of 752 gene knockouts but were able to annotate cellular phenotypes for only 331 genes, as detailed in their dataset. Of these 331 genes, 327 overlap with our dataset. We compared $\mathbb{P}$ for these 327 overlapping genes with the number of phenotypes reported in their study for each gene to observe a significant correlation (Spearman's ρ=0.55, p-value=8.48×10^{-28}; Pearson's r=0.53, p-value=1.44×10^{-24}).

Finally, Green *et al.* [6] analyzed 503 gene knockouts and searched for cellular phenotypes related to the germ layer or morphogenesis. They provided a dataset of associations between those 503 genes and phenotypes IDs from the WormBase (15 IDs related to germ layer and 15 to morphogenesis). The association score was the number of embryos that had that gene knockout and showed the phenotype. We find 475 genes in common with our dataset. We defined *their* pleiotropy value for each gene as

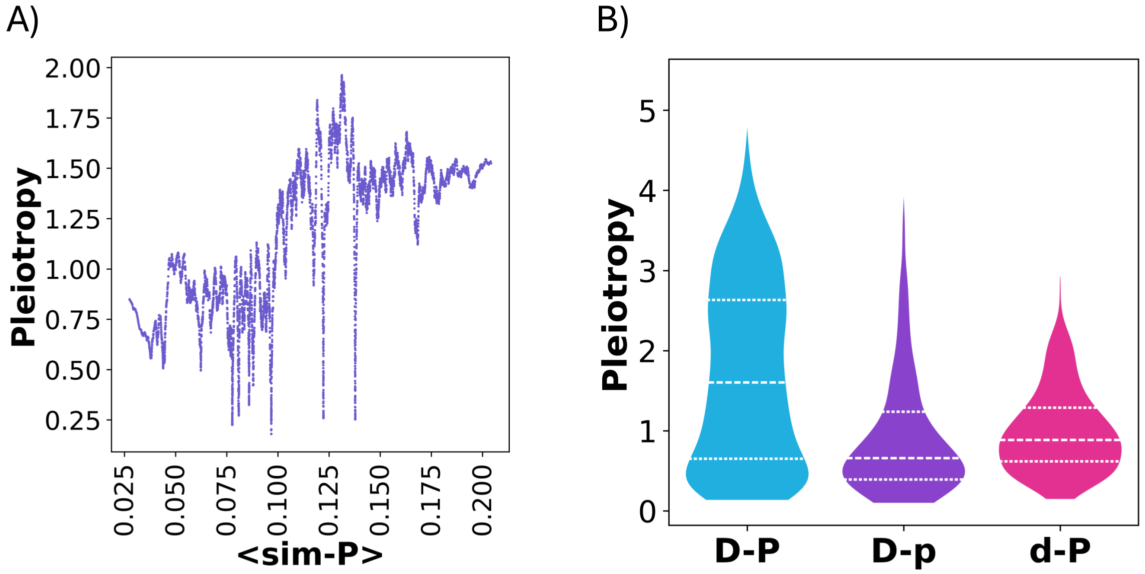

Fig O. Relation between pleiotropy and the $\mathcal{D}$–$\mathcal{P}$ rule. A) Pleiotropy *vs.* $\langle sim_{\mathrm{P}} \rangle$. Sliding window = 100. **B)** Pleiotropy distributions of `D-P`, `D-p` and `d-P` genes.

the sum of the fractions of embryos in which the knockout of that gene was associated with specific phenotypes. A gene was classified as pleiotropic if its knockout significantly impacted a large proportion of embryos. However, we observed no correlation between that measure of pleiotropy and our $\mathbb{P}$ value.

Coarse interpretation of the $\mathcal{D}$–$\mathcal{P}$ rule

The analysis of significant KS statistics allowed us to establish connections between phenotypes and cell types (Methods, main text). For instance, Fig 4B in the main text connects NMF phenotype component #49 with ciliated amphid neurons. Similarly, Fig PA associates phenotype component #82 with body wall muscle, while Fig PB links NMF phenotype component #5 with excretory cells.

Fig QA illustrates the distribution of the number of cell types mediating phenotypes, ranging from approximately 10 to 70 out of a total of 137 cell types. Fig QB presents the inverse perspective, showing how many phenotypes are mediated by each cell type (total of 100 phenotypes). Additionally, note that the higher the KS statistic (ranging from 0 to 1), the stronger the association between a cell type and an NMF-derived phenotype. The distribution of this statistic for all significant cases (p-value>0.0001, Fig 4C of the main text) is displayed in Fig QC.

To identify cell types with a high impact on a larger number of phenotypes (and vice versa), we selected associations where the KS statistic exceeded the 75th percentile and counted these for each cell type and NMF phenotype (S9 Table). The cell types mediating the greatest number of phenotypes are primarily found in clusters 1 and 5 (Fig RA, orange and brown clusters, respectively; this corresponds to the top-down orientation Fig 4C, main text; see also next section). Within cluster 1, the top cell types are precursor cells, while cluster 5 includes intestinal cells, M cells, glia, and pharyngeal muscle.

Moreover, NMF phenotypes strongly associated with many cell types include those related to chemosen-

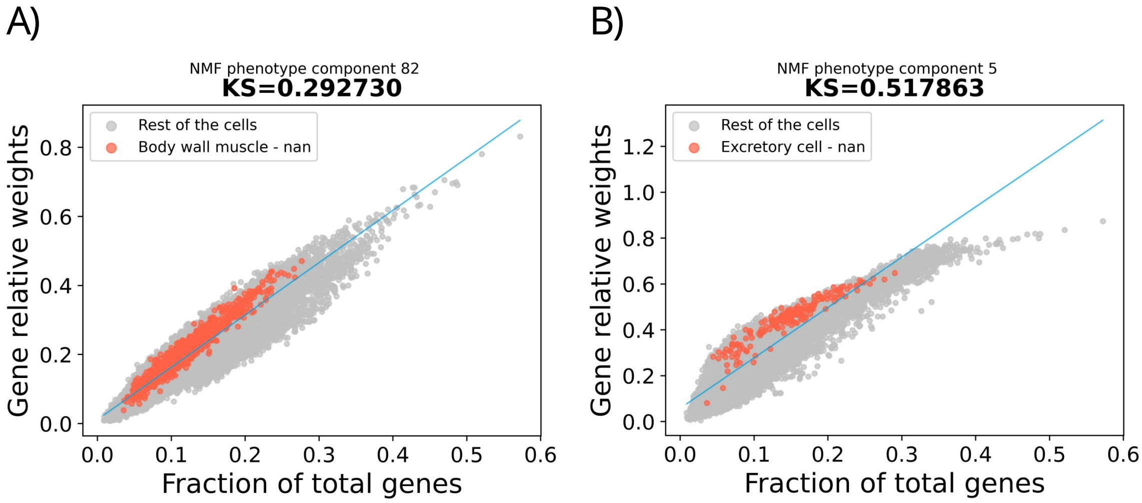

Fig P. Associations between a NMF phenotype and a cell type. Other examples of identifying mediator cells for NMF-derived phenotypes; A) phenotype #82 (body wall muscle cells, KS ≈ 0.3) and B) phenotype #5 (excretory cells, KS ≈ 0.5).

sory behavior, organ morphology, cytoskeleton organization, and drug resistance. These phenotypes belong to the red, orange, and purple clusters in the top dendrogram (Fig 4C, main text). The most relevant cell types are neurons (red and orange clusters) and neuronal precursors (purple cluster), with the abundance of distinct neuron annotations driving more significant nervous system-related associations with high KS values.

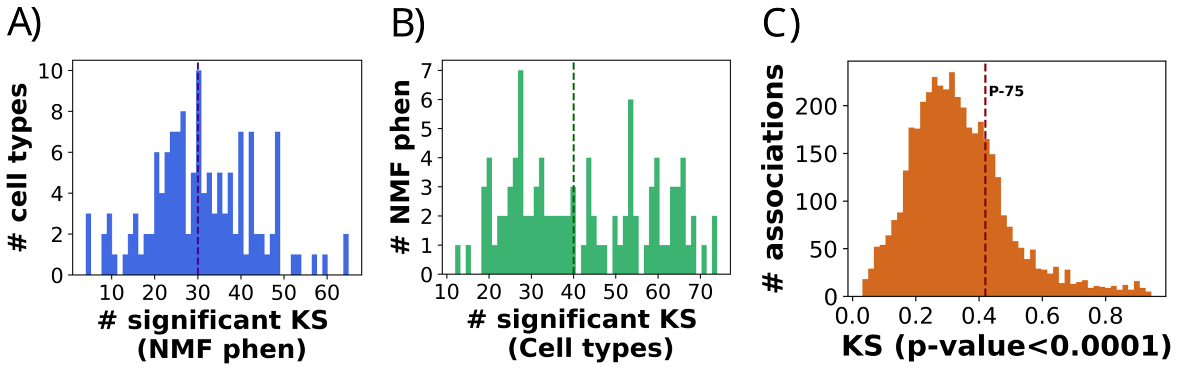

Fig Q. KS value distributions. A) Number of significant associated cell types per NMF component. B) Number of significant associated NMF components per cell types. The dashed line in plots A and B represents the median. C) Distribution of all the significant KS values (p-value < 0.0001). The dashed line indicates the percentile 75th.

Association between lineages and cell types belonging to each cluster

As discused before, the KS association between cell types and NMF components and the posterior clustering allows the cells to be grouped together in more generalized groups that identify the same kind of cell types: such as precursor and parent cell types, cell types related to nervous system and cell types related to different anatomical parts (pharynx, hypodermis, intestine, body wall). These clusters are shown in the dendrogram of Fig 4C of the main text and in Fig RA.

We also computed the percentage of cells in each cluster associated with each *C. elegans* lineage. By identifying the proportion of each cell type labeled with a lineage and averaging these proportions within

clusters, we determined the overall percentage of cells per cluster linked to each lineage. The results are shown in Fig RB.

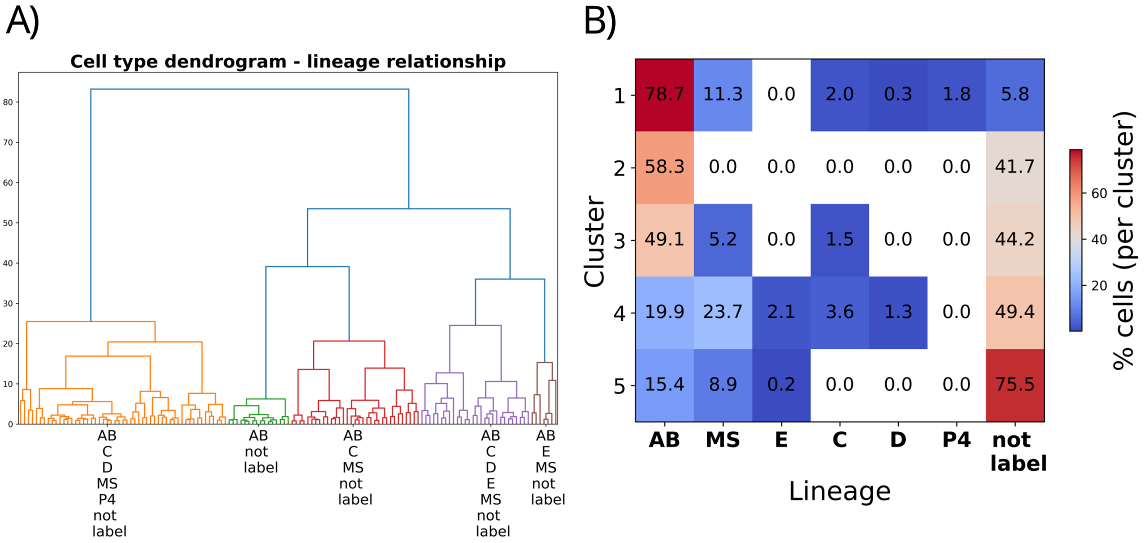

Fig R. Association between lineages and cell types' clusters. A) Dendrogram of the cell types. This dendrogram is obtained from the KS association between NMF components and cell types. It is also shown in Fig 4B of the main text. We find 5 labeled clusters. B) Percentage of cells of each cluster associated with each lineage.

The $\mathcal{D}$–$\mathcal{P}$ rule using a systematic dataset

We have also reanalyzed the $\mathcal{D}$–$\mathcal{P}$ rule and recalculated pleiotropy using a more systematic dataset. To this aim, we retained only those gene–phenotype associations annotated as "RNAi." These annotations can originate from different types of studies, including genome-wide RNAi, high-throughput RNAi, or targeted RNAi screens. To further ensure that the RNAi data used came from systematic or semi-systematic studies, we examined the number of distinct genes associated with phenotypes in each publication linked to the annotations.

As a filtering criterion, we retained only those publications that reported gene–phenotype associations for more than 100 genes, under the assumption that such studies are more likely to be large-scale and unbiased. This process resulted in a final set of 6,350 genes. Of these, 5,872 genes overlapped with the genes for which we have developmental similarity data. Using this restricted set of genes and phenotypes, we computed a new gene–phenotype association matrix. We also retrieved the developmental expression patterns of the corresponding genes and computed the similarities necessary to evaluate the $\mathcal{D}$–$\mathcal{P}$ rule. The signal appears similar to that obtained when using the full set of genes. (Spearman correlation between average similarities per gene: subset: $\rho = 0.308$, p-value=3.35×10^{-129}; full data set: $\rho = 0.309$, p-value=8.28×10^{-182}, Fig S).

As a complementary test, we validated the rule with this same systemic subset while controlling for the number of phenotypic components associated with each gene. Specifically, we counted the number of weights $w_i >$ per gene in the W matrix. If similarity were driven solely by this third variable, the partial correlation would considerably decrease. However, the $\mathcal{D}$–$\mathcal{P}$ rule remains significant (Spearman's

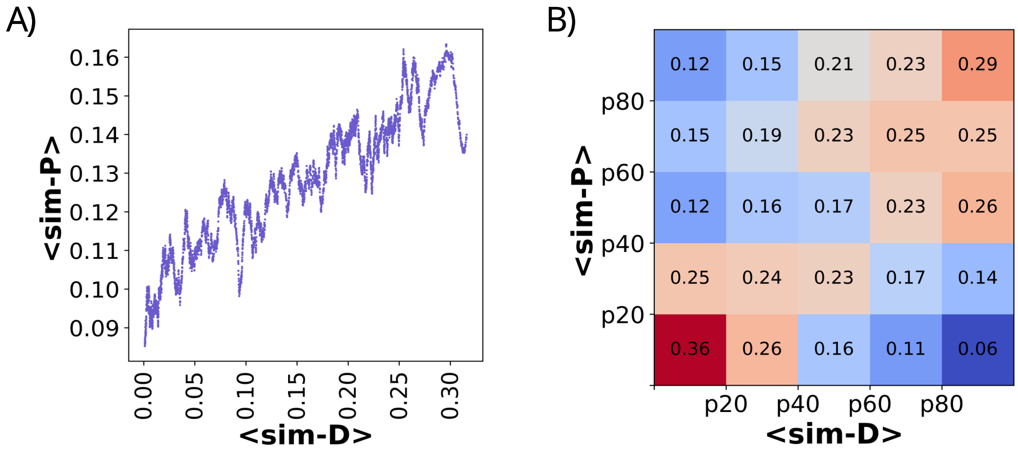

Fig S. The $\mathcal{D}$–$\mathcal{P}$ rule with a systematic dataset. A) Proportionality between $\langle sim_{\mathrm{D}} \rangle$ and $\langle sim_{\mathrm{P}} \rangle$ what highlights the $\mathcal{D}$–$\mathcal{P}$ rule: "similar developmental trajectories lead to similar phenotypes". Plot is a slidding window of the systematic data (windows size = 100). B) For each developmental similarity percentile group, we show the distribution of phenotypic percentiles (columns sum to 1). Deviations from uniformity indicate departures from the $\mathcal{D}$–$\mathcal{P}$ rule; see main text.

partial ρ=0.29, p-value=4.21×10^{-64}).

Finally, we used this very same dataset to calculate the corresponding pleiotropy by summing the weights associated with the matrix W obtained by applying NMF to the new gene–phenotype association matrix. We observed a strong correlation between the new and previously computed pleiotropy estimates (Pearson's $r = 0.83$, p-value=0).

The $\mathcal{D}$–$\mathcal{P}$ rule under tightly controlled, resolution-matched conditions

We independently validated our $\mathcal{D}$–$\mathcal{P}$ rule using a dataset from Xiao *et al.* [5], which provides systematic measurements of *embryonic* phenotypes at single-cell resolution following perturbation of 752 genes. The phenotypes correspond to cell cycle length, division asynchrony, sibling cell division asymmetry, CND-1 expression status (a neuronal fate marker), division angle, and relative cell position. These measures are well aligned with the embryonic single-cell transcriptomic data used in this study, thereby allowing us to test the generalizability of our findings.

To construct the gene–phenotype association matrix, we included cases where the mutant phenotype showed a significant deviation from the expected value observed in control (wild-type) embryos. Significance was determined by converting z scores to two-tailed p values, then adjusting them for multiple testing to q values. We retained only associations with $q < 0.01$ (and, for CND-1 expression, entries explicitly marked as 1 or −1). For each gene, if any knockdown embryo exhibited a significant association with a specific cell/lineage–phenotype combination, we assigned a value of 1 to that matrix entry; otherwise, we assigned a 0. Note that following this approach, we identified 658 genes affecting at least one of the six phenotypes (in any cell), compared to the 331 genes reported by Xiao *et al.* [2022] on their website (dulab.genetics.ac.cn/single-cell-phenomics) and that we used for pleiotropy analysis. Both phenotype counts strongly correlate (Spearman's ρ=0.84, p-value=8.09×10^{-90}).

Regarding gene expression during development, Xiao *et al.* [5] measured phenotypes up to the 350-cell stage, which corresponds to approximately 7 hours post-fertilization (420 minutes). Since only genes expressed within this developmental window can contribute to the observed phenotypes, we restricted our original developmental expression matrix (organized by cell type and time) to time points up to 420 minutes.

Armed with these two matrices, we calculated the average developmental and phenotypic similarities for each gene as in the main text. We observed a significant correlation between the two (Spearman's ρ=0.180, p-value=1.10×10^{-5}, Fig T), supporting the validity of the rule.

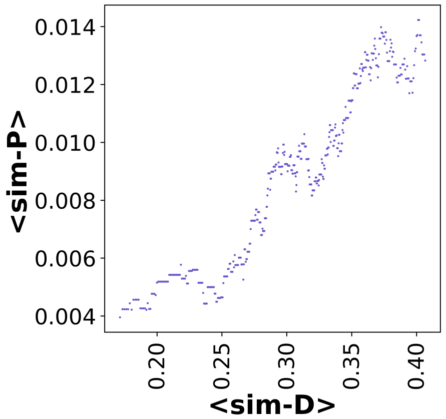

Fig T. The $\mathcal{D}$–$\mathcal{P}$ **rule using systematically measured embryonic phenotypes at single-cell resolution following gene perturbation.** We confirm our findings using a more controlled and resolution-matched dataset from Xiao *et al.* [5]. We observed a proportional relationship between $\langle sim_{\mathrm{D}}\rangle$ and $\langle sim_{\mathrm{P}}\rangle$, highlighting the presence of the rule. The plot shows a sliding window analysis over the full dataset (window size = 100).

The $\mathcal{D}$–$\mathcal{P}$ rule and its coarse interpretation in zebrafish

To assess the cross-species applicability of our approach, we applied it to zebrafish (*Danio rerio*). We used scRNA-seq data from Lange *et al.* [7] in which developmental gene expression was organized by cell types and temporal stages –from 10 hours post-fertilization (hpf) to 10 days post-fertilization (dpf). The phenotype annotations are obtained from the Zebrafish Information Network (ZFIN, [8]), where phenotypes are described using anatomical terms.

To construct the developmental gene expression matrix, we quantified, for each gene, the number of cells in which it was expressed within a given cell type and embryonic time point. This count was then normalized by the total number of cells identified for that specific cell type and time point. To construct the gene–phenotype association matrix, we used available zebrafish datasets linking phenotypes to developmental stages (the stages at which phenotypes were observed or measured). We excluded gene–phenotype associations in which the phenotype endpoint occurred before 10hpf, corresponding to the gastrula phase (specifically, the 50% epiboly stage). Only three phenotypes ended prior to this stage: blastodisc, fertilized egg, and angiogenic sprout. Of the initial 136,106 associations, 198 were removed.

The $\mathcal{D}$–$\mathcal{P}$ rule held true in zebrafish, although the correlation was weaker than in *C. elegans* (Fig U, Spearman's ρ=0.14, p-value=7.42×10^{-24}). We observed a higher average pairwise similarity among gene expression profiles across developmental stages in zebrafish. This pattern might show a biological

difference. In *C. elegans*, cells follow a fixed lineage, which means they turn on specific genes closely linked to their division history, leading to more differences in gene activity. In zebrafish, development is more flexible, and cells primarily determine their fate based on signals from their surroundings rather than their lineage. Because of this, many genes are used in different cell types, making cells more similar on average during development.

However, this pattern could also be partially explained by technical factors. The zebrafish data set had a wider cell coverage, increasing the likelihood of detecting gene expression in more cell types and stages. Furthermore, only anatomical phenotypes are available. These factors represent important limitations when comparing developmental trajectories and gene–phenotype relationships across species.

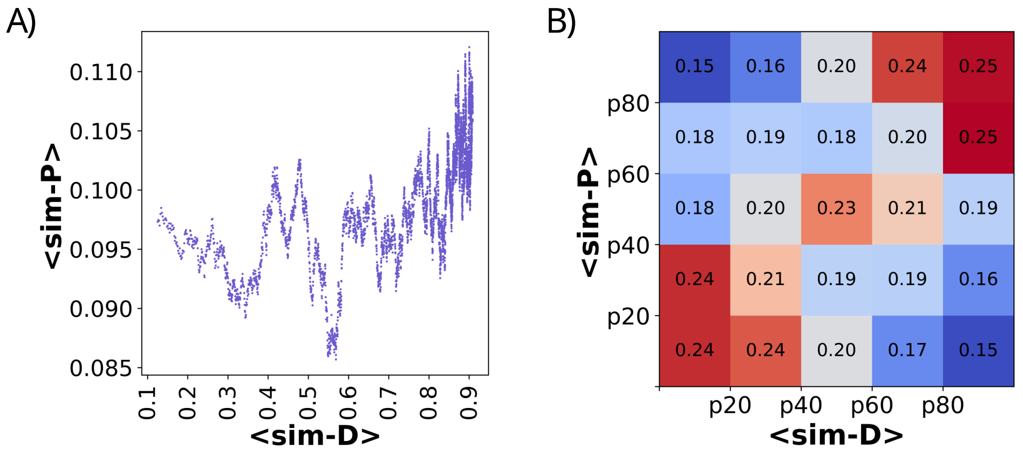

Fig U. The $\mathcal{D}$–$\mathcal{P}$ rule in zebrafish. A) Proportionality between $\langle sim_{\mathrm{D}} \rangle$ and $\langle sim_{\mathrm{P}} \rangle$ what highlights the $\mathcal{D}$–$\mathcal{P}$ rule: "similar developmental trajectories lead to similar phenotypes". Plot is a sliding window of the zebrafish data (windows size = 100). B) For each developmental similarity percentile group, we show the distribution of phenotypic percentiles (columns sum to 1). Deviations from uniformity indicate departures from the $\mathcal{D}$–$\mathcal{P}$ rule.

We also studied deviations from the rule. As in *C. elegans*, we classified zebrafish genes into three categories based on the $\mathcal{D}$–$\mathcal{P}$ rule (Fig VA): `D-P` genes ($n = 790$), with high similarity in both developmental expression and phenotype; `D-p` genes ($n = 136$), with high developmental similarity but low phenotypic similarity; and `d-P` genes ($n = 137$), with low developmental similarity but high phenotypic similarity. `D-P` and `D-p` genes tend to exhibit ubiquitous developmental expression, while `d-P` genes show more diverse and specific patterns (Fig VB). In terms of pleiotropy, `D-P` genes are the most pleiotropic, followed by `d-P` and then `D-p` genes (Fig VC). Phenotype enrichment analysis based on the original gene–phenotype matrix supports these trends (S10 Table). `D-P` genes are enriched in 116 phenotypes spanning multiple levels of anatomical organization, including major systems such as the nervous system (e.g., 'brain', 'neurons'), craniofacial skeleton (e.g., 'pharyngeal arch cartilage'), and sensory organs (e.g., 'eye', 'visual system'). `d-P` genes are enriched in 11 phenotypes, with 10 overlapping those of `D-P` genes, primarily related to sensory and nervous system development in the head and eye regions. In contrast, `D-p` genes show enrichment in 5 phenotypes, involving only a small subset of genes primarily associated with the hematopoietic and circulatory systems. In summary, `D-P` genes are broadly expressed and highly pleiotropic, often influencing major anatomical systems; `d-P` genes, while more restricted in expression, still affect key anatomical domains; and `D-p` genes, despite their broad expression, are less pleiotropic and tend to impact more functionally independent systems such as the circulatory system.

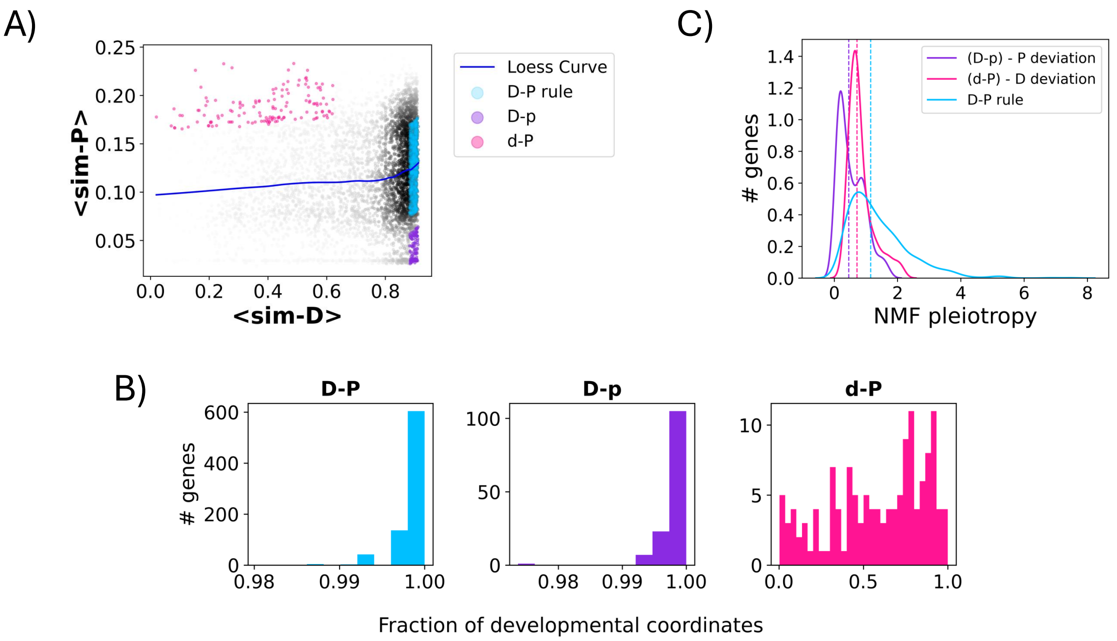

Fig V. D-P rule deviations in zebrafish. A) $\langle sim_{\mathrm{D}} \rangle$ *vs.* $\langle sim_{\mathrm{P}} \rangle$ per gene. The blue line is the Loess curve. We define three sets of genes to analyze: `D-P` genes (blue), `D-p` genes (purple) and `d-P` genes (pink). B) Distributions of non-zero developmental coordinates. C) Kernel density estimates of pleiotropic score distributions for `D-P`, `d-P`, and `D-p` genes. The x-axis represents pleiotropy scores, and the y-axis indicates the fraction of genes in each group. Dashed lines show medians.

Finally, the study of the *coarse* interpretation of the $\mathcal{D}$–$\mathcal{P}$ rule revealed significant relationships between phenotypic components derived from NMF and specific cell types (Fig WA; S11 Table). We focused on the highest KS scores, which highlighted three strong associations: NMF cluster 2 (orange) with cell type cluster 1 (red); NMF cluster 1 (red) with cell type cluster 2 (orange); and NMF cluster 4 (blue) with cell type cluster 5 (magenta).

The first cell type cluster (red) comprises early embryonic structures rather than mature cell types, including the ‘floor plate’, ‘aortic arch’, ‘artery’, ‘vein’, ‘blood vasculature’, and ‘ganglion’. This group closely aligns with NMF cluster 2 (orange), whose associated original phenotypes are related to cardiovascular elements (‘blood vessel’, ‘vasculature’, ‘axial vasculature’, ‘trunk vasculature’).

NMF cluster 1 (red) is strongly associated with cell type cluster 2 (orange). We find that the most influential original phenotypes include ‘epithelium’, ‘musculature system’, ‘muscle’, and ‘surface structure’. The cell type cluster consists of muscle-related cells such as ‘cardiac muscle cell’, ‘muscle pioneer’, ‘adaxial cell’, ‘somite’, and ‘lateral plate mesoderm’ and epithelial-related cells like those in the ‘mesenchyme pectoral fin’ and ‘head mesenchyme’.

The third association connects NMF cluster 4 (blue) with cell type cluster 5 (magenta). The associated phenotypes are primarily related to the sensory systems, particularly the visual and nervous systems. Specific examples include the ‘brain’, ‘eye’, ‘spinal cord’, and ‘retina’. The corresponding cell types are involved in sensory processing –such as ‘eye photoreceptor cells’, ‘retinal rod cells’, ‘retinal cone cells’, and ‘amacrine cells’– and in the development of the sensory system, including structures like the ‘forebrain’ and ‘midbrain interneurons’.

As observed, the first clusters of phenotypes and cell types correspond to earlier developmental stages, while the later clusters are associated with more specialized phenotypes and anatomically defined

structures characteristic of advanced system development.

In Figs WB-WD, we highlight specific examples of strong cell type–phenotype associations. Among all NMF components, component 4 from NMF cluster 4 (blue) shows the highest number of cell type associations with KS values above the 75th percentile of the distribution (Fig WB). The top-weighted original phenotypes in this component include ‘electrically active cell’, ‘electrically responsive cell’, and ‘neuron’, and is strongly associated with ‘primary neuron’ and ‘forebrain’. Similarly, component 41, also part of NMF cluster 4 (blue) (Fig WC), is dominated by phenotypes such as ‘photoreceptor cell’, ‘retinal photoreceptor layer’, and ‘retinal outer nuclear layer’, and is associated with ‘eye photoreceptor cell’ and ‘retinal cone cell’. Furthermore, component 16 of NMF cluster 1 (red) is enriched in more general epithelial-related phenotypes, such as ‘epithelium’, ‘epithelial cell’ and ‘portion of tissue’ (Fig WD), and is associated with ‘pharyngeal arch 3-7’ and ‘pectoral fin musculature’.

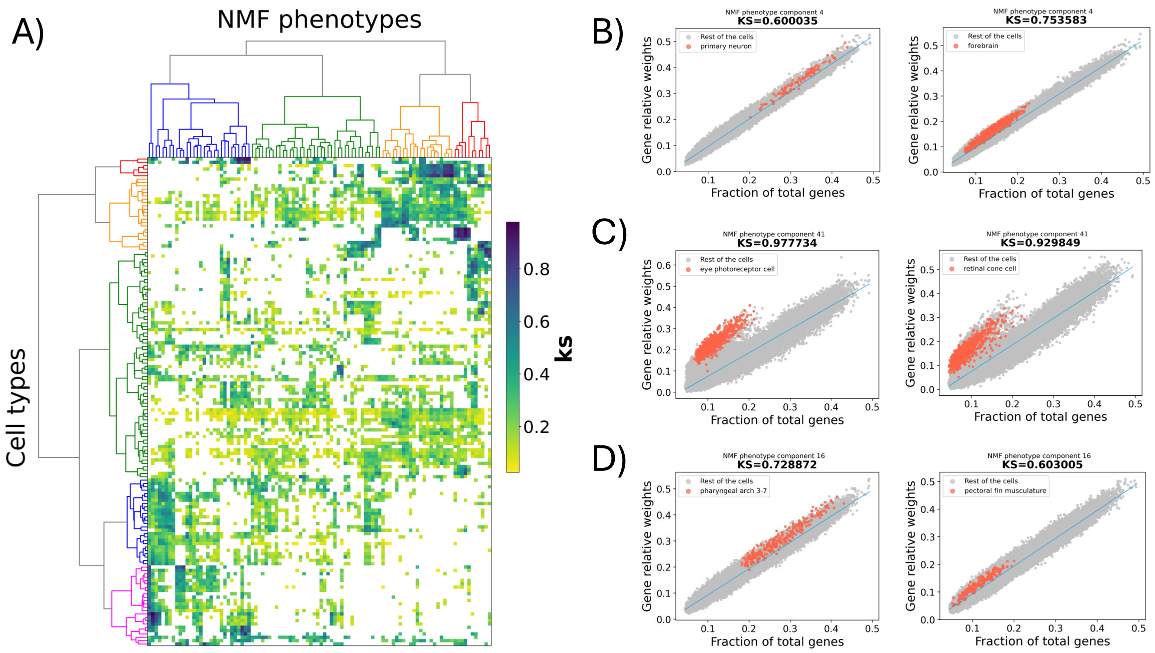

Fig W. Coarse interpretation of the rule in zebrafish. A) Heatmap that maps NMF phenotype components to specific cell types. Each association in the heatmap is colored according to the KS statistic, with all displayed values corresponding to statistically significant associations (p-value < 0.0001). B, C, D) Specific associations between NMF phenotypes and cell types. B) NMF component 4 is associated with‘primary neuron’ and ‘forebrain’. C) NMF component 41 is linked to ‘eye photoreceptor cell’ and ‘retinal cone cell’. D) NMF component 16 is associated with ‘pharyngeal arch 3–7’ and ‘pectoral fin musculature’.

References

[1] Packer JS, Zhu Q, Huynh C, Sivaramakrishnan P, Preston E, Dueck H, et al. A lineage-resolved molecular atlas of *C. elegans* embryogenesis at single-cell resolution. Science. 2019;365(6459):eaax1971. doi:10.1126/science.aax1971.

[2] Schindelman G, Fernandes JS, Bastiani CA, Yook K, Sternberg PW. Worm Phenotype Ontology: Integrating phenotype data within and beyond the *C. elegans* community. BMC Bioinformatics. 2011;12(1):32. doi:10.1186/1471-2105-12-32.

[3] Lee DD, Seung HS. Learning the parts of objects by non-negative matrix factorization. Nature. 1999;401(6755):788–791. doi:10.1038/44565.

[4] Zou L, Sriswasdi S, Ross B, Missiuro PV, Liu J, Ge H. Systematic Analysis of Pleiotropy in *C. elegans* Early Embryogenesis. PLoS Comput Biol. 2008;4(2):e1000003. doi:10.1371/journal.pcbi.1000003.

[5] Xiao L, Fan D, Qi H, Cong Y, Du Z. Defect-buffering cellular plasticity increases robustness of metazoan embryogenesis. Cell Syst. 2022;13(8):615–630.e9. doi:10.1016/j.cels.2022.07.001.

[6] Green RA, Khaliullin RN, Zhao Z, Ochoa SD, Hendel JM, Chow TL, et al. Automated profiling of gene function during embryonic development. Cell. 2024;187(12):3141–3160.e23. doi:10.1016/j.cell.2024.04.012.

[7] Lange M, Granados A, VijayKumar S, Bragantini J, Ancheta S, Kim YJ, et al. A multimodal zebrafish developmental atlas reveals the state-transition dynamics of late-vertebrate pluripotent axial progenitors. Cell. 2024;187(23):6742–6759.e17. doi:10.1016/j.cell.2024.09.047.

[8] Bradford YM, Van Slyke CE, Ruzicka L, Singer A, Eagle A, Fashena D, et al. Zebrafish Information Network, the knowledgebase for *Danio rerio* research. Genetics. 2022;220(4):iyac016. doi:10.1093/genetics/iyac016.
