# Supplementary material for: Cell atlases and the developmental foundations of the phenotype
Source: PLoS Comput Biol. 2026 Feb 9;22(2):e1013944. doi: 10.1371/journal.pcbi.1013944 (PMC12904592; doi:10.1371/journal.pcbi.1013944)
Supplement: Attachment — Submitted filename: Lou_etal_rebuttal.pdf [file pcbi.1013944.s013.pdf]

Reviewer #1:

Comments

1. Lack of conceptual novelty and predictable findings
The central idea that developmental gene expression correlates with phenotypic outcomes is well established in developmental biology and systems genetics. While this study provides a formal framework to quantify that relationship, the main conclusions are largely expected. Previous studies have explored similar genotype–phenotype relationships using expression atlases and phenotype ontologies. The manuscript does not sufficiently distinguish its contribution from this existing body of work. Moreover, many results, such as the observation that broadly expressed "housekeeping" genes tend to be pleiotropic and associated with systemic phenotypes, are intuitive and consistent with known biology. Although the analysis is technically sound and clearly presented, the findings are largely confirmatory and lack mechanistic novelty.

To our knowledge, relatively few studies have explicitly combined *cellular-resolution* expression atlases with *controlled vocabularies* such as the Worm Phenotype Ontology to examine development–phenotype relationships in this way. Projects like the Human Cell Atlas and the mouse Tabula Muris have annotated cell types across tissues, sometimes mapping to anatomical ontologies (like Uberon or the Cell Ontology). But these mostly capture structure, not phenotypic function or variation. One could argue that the emerging field of *single-cell phylo-transcriptomics* is the closest in spirit to our approach (e.g., https://doi.org/10.1016/j.tig.2024.02.005), where the goal is to compare developmental gene expression trajectories across species to assess whether similar activation patterns support shared phenotypes or conserved anatomical structures. This perspective aligns with our formulation of the Development–to–Phenotype (D–P) rule.

The reviewer notes that both the general association between gene expression and phenotype, and some of our specific findings –such as the pleiotropy of broadly expressed "housekeeping" genes– may be expected. While we acknowledge that these ideas have been discussed in prior literature, we consider it is not a weakness but a strength to now be able to quantify and test these hypotheses with modern datasets. For example, while von Baer's ideas presented in the nineteenth century that morphological differences between species increase as development advances it is only recently that we have been able to start testing them with the use of *bulk* transcriptomes across developmental stages in several species (e.g., https://doi.org/10.1038/s41586-019-1338-5).

With respect to mechanistic novelty, a central focus of our work is to examine where the D–P rule fails, and to understand which genes violate the expected expression–phenotype correlation, under what developmental conditions, and with what functional consequences. These exceptions are analyzed extensively in the Supplement, including phenotype and GO enrichment analyses of outlier genes (Tables S4–S5), as well as parallel analyses for pleiotropic genes (Tables S6–S7). Moreover, our coarse-grained developmental coordinate analysis introduces a more nuanced, cell type–resolved view of phenotypic influence, highlighting specific developmental contexts that disproportionately shape phenotypic outcomes (Supplement and Tables S8-S9; note that we extended the analysis of the relationship between cell type and phenotype in a new Table S9). In this way, the study provides mechanistic insight into how and where the D–P rule breaks down,

what pleiotropy looks like in a spatiotemporal context, and which cells are disproportionately involved –insights that are not accessible through bulk-level or annotation-only approaches.

We have revised the manuscript to better articulate earlier contributions and to clarify how our framework builds upon, but is distinct from, previous studies.

2. Oversimplification of phenotype emergence

The D–P rule rests on the assumption that similar developmental expression patterns should lead to similar phenotypic outcomes. This overlooks the inherent complexity of how phenotypes arise. Phenotypes are often emergent, context-dependent, and shaped by subtle primary defects that manifest as divergent secondary traits. In many cases, so-called "phenotypes" are higher-order or secondary outcomes that arise from cascades of subtle primary molecular or cellular defects. Two genes may cause seemingly similar organismal phenotypes (e.g., uncoordinated movement) through entirely different mechanisms and cell types (1). Conversely, two genes with very similar roles in development may lead to divergent or even undetectable phenotypes depending on buffering mechanisms, measurement resolution, or stage-specific compensation (2).

Additionally, gene redundancy and network buffering may prevent any observable phenotype from emerging, even when a gene has important developmental roles. A gene with specific and important developmental expression may not produce any overt phenotype when perturbed, due to functional compensation by paralogs or pathway-level redundancy. These "no phenotype" cases are especially problematic in datasets like the WPO, where negative results are underrepresented and often not systematically reported.

These biological realities make it unreasonable to expect consistent phenotypic similarity based solely on expression profiles. The authors should address these limitations more explicitly, and consider reframing the D–P rule as a probabilistic tendency rather than a deterministic rule. Incorporating measures of phenotype specificity or annotation confidence could help mitigate these issues.

We fully agree that phenotypes are often emergent, context-dependent, and shaped by layers of developmental and physiological interactions, including buffering, redundancy, and compensatory mechanisms. Far from ignoring this complexity, we highlighted it *explicitly* in the manuscript. As argued in the Introduction (previous version):

"*Although this question is necessarily broad –development both constrains (Alberch [1980]) and diversifies (West-Eberhard [2003]) phenotypes– we simplify it to a quantitative evaluation of a fundamental "rule" that … While this is a simplification of development's full complexity within the genotype-to-phenotype (GP) map, it offers an approach we can rigorously evaluate*".

In this light, the D–P rule is not intended as a deterministic claim, but as a statistical trend –a hypothesis that developmental similarity, in general, correlates with organismal phenotypic similarity, while allowing for numerous and biologically meaningful exceptions (we made clear in the abstract that the rule applies *on average*). We now make this point more explicit in both the Introduction and Discussion.

Moreover, the reviewer's comments on buffering, redundancy, etc., align directly with the mechanisms we discuss in our analysis of *deviations* from the D-P rule, which we interpret as signatures of phenotypic degeneracy (Edelman & Gally, 2001) and developmental divergence (Carroll et al., 2005). These deviations are not treated as noise or exceptions to be dismissed, but rather as valuable cases for understanding why equivalent developmental expression may not translate into similar phenotypes (or *vice versa*); precisely the biological complexity the reviewer raises. In this way, the second half of our results moves beyond confirming a statistical trend to exploring its mechanistic boundaries.

Regarding the concern about underreporting of negative phenotypes in the Worm Phenotype Ontology (WPO), we completely agree. We now explicitly acknowledge in the description of this data (Materials and methods) that the lack of an annotated phenotype does not imply a lack of functional importance. This limitation -stemming from detection thresholds, stage-specific effects, and genetic redundancy- is one reason why we chose to work with continuous phenotypic similarity scores rather than binary phenotypic presence/absence. We also mitigate some of the redundancy in the WPO dataset through dimensionality reduction using non-negative matrix factorization, allowing us to infer broader patterns of phenotype similarity that might otherwise be hidden.

In summary, we respectfully consider that the reviewer's critique, while valid in highlighting the complexity of phenotype emergence, is not in conflict with our approach. On the contrary, that complexity is what motivates our framework: the D–P rule serves as a testable statistical hypothesis whose *strength lies not only in the signal it reveals but also in the biological insights uncovered when it breaks down*. We have revised the manuscript to better communicate this framing and hope that this clarifies the conceptual intent and contribution of our work.

<u>3. Resolution mismatch between datasets</u>

There is a fundamental mismatch between the resolution of the gene expression and phenotype data. The expression data are derived from a single high-resolution embryonic single-cell RNA-seq dataset, while the WPO phenotypes are compiled from heterogeneous sources spanning multiple life stages and experimental conditions. This discrepancy introduces substantial noise. Genes with broad phenotype annotations may reflect research bias rather than biological pleiotropy, while genes with few annotations may be under-studied rather than functionally narrow.

To strengthen the analysis, the authors should stratify phenotypes by source or method (e.g., curated vs. high-throughput RNAi), normalize for annotation density, and consider focusing on more standardized datasets, such as genome-wide RNAi screens. Benchmarking against a high-confidence gene-phenotype subset could also help validate key conclusions.

<u>Bias</u>.

We recognize that annotation-based pleiotropy can reflect research bias, as more extensively studied genes tend to have more recorded phenotype. However, this does not mean that such pleiotropy is not biologically meaningful. For example, Stoeger *et al.* (https://doi.org/10.1371/journal.pbio.2006643) studied human gene characteristics in relation to publication frequency and found that one of the main features of highly studied genes is high RNA

and protein levels across multiple tissues, which reflects their fundamental biological roles. Then, genes with broad phenotype annotations, although more frequently studied, also reflect underlying biology, often linked to housekeeping tasks and widespread expression across tissues. For genes that have been studied at least once and consistently show no evidence of general biological functions, it is plausible to assume that they may indeed lack such broad roles. Our concern regarding research bias applies most strongly to genes whose expression has not yet been detected due to technical challenges, rather than to those already studied but found to have narrow or context-specific functions.

In fact, our pleiotropy measure is consistent with the findings discussed above. In the manuscript, in the pleiotropy section (previous version):

"*The results showed that pleiotropic genes are expressed in significantly more cells than expected. In contrast, non-pleiotropic genes (those below the 5th percentile of the distribution, Figure 3A) are expressed in far fewer cells and show no notable enrichment for phenotypes or GO terms. Pleiotropic genes, however, are strongly associated with systemic phenotypes and functions (Table S6-S7)*".

In the Supplement (page 13, within the Pleiotropy section):

"*With respect to the GO terms, the pleiotropic genes are enriched in a high number of them including: 'intracellular anatomical structure', 'cellular process', 'anatomical structure development', 'cellular component organization or biogenesis' and 'positive regulation of biological process'. Non-pleiotropic genes are enriched in just 6 terms such as 'zinc ion binding' and 'DNA-binding transcription factor activity'. Those terms are associated with a small subset of non-pleiotropic genes.*"

Moreover, we observed a correlation between the number of publications and our pleiotropy measure (Fig. 1 for reviewer #1 below, $r = 0.819$, p = 0), although some highly studied genes fall below this trend. Our NMF analysis revealed that many of the phenotypes associated with these genes are similar and tend to cluster within the same NMF components. By capturing this redundancy in the phenotype ontology space, NMF helps mitigate the impact of research bias and improves biological interpretability by retaining only the most informative phenotypic components. Note also that NMF tends to assign non-zero weights only where necessary, which downweights noisy or overly broad profiles that might come from high annotation density. Moreover, heavily annotated genes often show inflated similarity simply because they share many terms by chance. NMF smooths that raw signal, so phenotype similarity is measured in terms of shared contribution to latent features, not just term overlap.

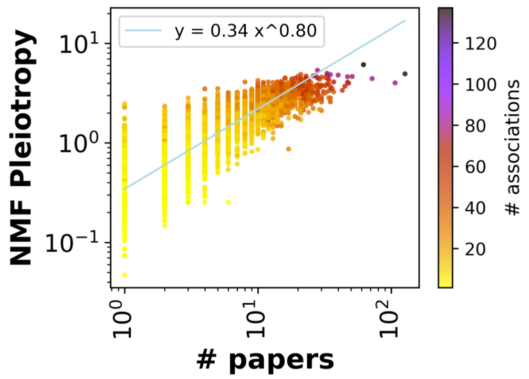

Figure 1 for reviewer #1. *Correlation between number of publications and NMF-based pleiotropy.*

Still, the observed correlation between pleiotropy and number of publications may reflect biological importance: Highly pleiotropic genes attract attention because they impact multiple biological systems, are involved in multiple diseases or traits and tools and data already exist for them (e.g., antibodies, cell lines). This leads to a rich-get-richer effect, where genes already known to be pleiotropic become more studied, and hence more pleiotropic functions are discovered (as discussed in https://doi.org/10.1371/journal.pbio.2006643).

Therefore, while we acknowledge that publication count is an important factor to consider when interpreting our results, we maintain that the genes identified as pleiotropic through our approach are biologically meaningful. We now include a brief comment of this in the discussion.

Systematic dataset.

On the other hand, we have also reanalyzed the D-P rule and recalculated a new pleiotropy score using only *genes and phenotypes derived from high-throughput RNAi experiments*, as recommended by the reviewer.

We thus retained only those gene–phenotype associations annotated as "RNAi." These annotations can originate from different types of studies, including *genome-wide* RNAi, *high-throughput* RNAi, or *targeted* RNAi screens. However, the annotation itself does not always specify the scale or scope of the experiment. Therefore, to further ensure that the RNAi data used came from systematic or semi-systematic studies, we examined the number of distinct genes associated with phenotypes in each publication linked to the annotations.

As a filtering criterion, we retained only those publications that reported gene–phenotype associations for more than 100 genes, under the assumption that such studies are more likely to be large-scale and unbiased. This process resulted in a final set of 6,350 genes. Of these, 5,872 genes overlapped with the genes for which we have developmental similarity data. Using this restricted set of genes and phenotypes, we computed a *new* gene–phenotype association matrix. We also retrieved the developmental expression patterns of the corresponding genes and computed the similarities necessary to evaluate the D-P rule.

Note that normalization by term density is not necessary in our case because the computation of similarities is based on cosine distance, which inherently normalizes for annotation density by

dividing the dot product of two vectors by the product of their norms. In doing so, it measures the angle between vectors rather than their absolute magnitudes, thereby mitigating the influence of differing annotation counts.

The D-P rule still holds. The signal appears similar to that obtained when using the full set of genes. (Correlation between average similarities per gene: <simD> and <simP> subset: Spearman's ρ = 0.308, p-value=3.35e-129; full data set: ρ = 0.309, p-value=8.28e-182)

As a complementary test, we validated the D-P rule with this same systemic subset while *controlling* for the number of phenotypic components associated with each gene. Specifically, we counted the number of weights $w_i>0$ per gene in the W matrix. If similarity were driven solely by this third variable, the partial correlation would considerably decrease. However, the D–P rule remains significant (Spearman's partial ρ=0.29, p-value=4.21e−64).

Finally, we used this very same dataset to calculate the corresponding pleiotropy by summing the weights associated with the matrix W obtained by applying NMF to the *new* gene–phenotype association matrix. We observed a strong correlation between the new and previously computed pleiotropy estimates (Pearson's r = 0.831, p-value=0, Figure 2 for reviewer #1, where genes with residuals exceeding two standard deviations are highlighted in orange –above, ~2%, n = 143 genes– and red –below, ~4%, n = 241 genes.

We now include this new analysis in the Discussion and Supplement (section *'The D–P rule using a systematic dataset'*).

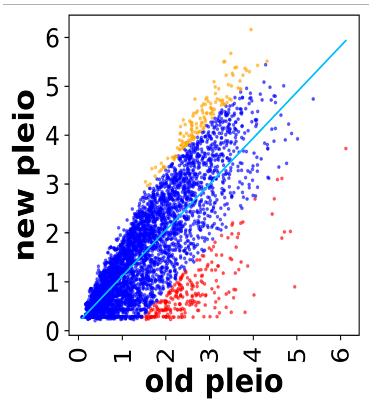

Figure 2 for reviewer #1. *Correlation between NMF-based pleiotropy with the full ('old') and systematic ('new') dataset.*

4. Temporal misalignment between expression and phenotype

The study uses embryonic gene expression data but compares it to phenotype annotations that include post-embryonic and adult stages. Many of the phenotypes analyzed likely arise from gene functions outside the developmental time window studied, weakening the claim that embryonic expression patterns alone predict phenotypic outcomes. The authors should consider restricting phenotype analysis to embryonic or early post-embryonic phenotypes, or at least stratifying phenotypes by developmental timing where possible. This limitation should also be discussed more explicitly in the manuscript.

We recognize the reviewer's concern that some phenotypes –particularly behavioral and reproductive traits– manifest post-embryonically and may reflect gene functions beyond the embryonic window captured in our dataset (mid-gastrulation to terminal differentiation). However, our central hypothesis is that *embryonic gene expression establishes foundational cellular and tissue-level programs that strongly influence downstream phenotypic outcomes*, even when these phenotypes emerge later in life.

Many adult phenotypes in *C. elegans* trace back to developmental patterning events during embryogenesis. For example, genes involved in specifying neuronal or muscle lineages, such as *unc-86* and *egl-5*, are expressed embryonically but give rise to defects observable only in adult behaviors or morphologies. Specifically, *unc-86* is expressed in neuroblast lineages during embryogenesis and is required for proper specification of HSN and mechanosensory neurons; mutations result in egg-laying defects and touch insensitivity in adults (https://doi.org/10.1093/emboj/19.14.3694). Similarly, *egl-5*patterns posterior fates during embryonic and larval development, yet mutants exhibit fused tail rays and mating defects only in L4/adult males (https://doi.org/10.1242/dev.120.9.2579).

Regarding the suggestion to restrict or stratify phenotypes by developmental timing: we would be very interested in performing such an analysis. In other model organisms' databases, such as zfin for zebrafish (https://zfin.org), this information is available: phenotype annotations can also refer to a developmental stage (i.e., the time when the phenotype was observed or measured). However, as far as we are aware, the phenotype annotations in wormbase do not provide systematic or consistent developmental-stage metadata specifying when a phenotype is observed or measured. This lack of temporal resolution limits our ability to implement the stratification the reviewer suggests.

We have now clarified these issues in the revised manuscript.

<u>5. Lack of experimental validation</u>
The entire study is computational and retrospective. Given the central hypothesis, experimental validation would add significant value. For example, the authors could use their similarity matrices to predict phenotypes of understudied genes and test a small number through RNAi perturbation, followed by phenotypic assays, ideally during embryogenesis, to match the expression data. Even partial validation of such predictions would greatly strengthen the paper and demonstrate that the D–P rule has practical utility beyond descriptive modeling.

We appreciate the reviewer's suggestion and agree that experimental validation would meaningfully extend the impact of this work. Indeed, using our similarity matrices to predict phenotypes of understudied genes and testing these via RNAi or other perturbation methods is a compelling next step.

However, this study was intentionally designed as a computational investigation, in line with the scope of *PLOS Computational Biology*, with the goal of identifying broad, genome-scale principles –specifically the statistical relationship between gene expression trajectories and phenotypic similarity (the D–P rule). As noted by the reviewer earlier, the rule is statistical in nature and reflects a global tendency, rather than a direct prediction of phenotypes for individual genes.

That said, we agree that future work could build on our pairwise similarity matrices to develop predictive models for gene-specific phenotypes. We now include a comment on the discussion

emphasizing how our framework generates testable hypotheses for functional validation. We believe that identifying such principles computationally is a critical step toward prioritizing targets for focused experimental studies, especially in systems like *C. elegans*, where large-scale perturbation resources exist.

6. Unvalidated cell-type–phenotype associations

The identification of specific cell types such as ASK neurons as key mediators of certain phenotypes (e.g., chemotaxis) is an interesting aspect of the analysis. However, this claim remains correlative. The authors could bolster this part of the study by perturbing genes enriched in these cell types and assessing the predicted behavioral outcomes. Cell-type–specific rescue experiments could also help establish causality.

Functional perturbation and rescue experiments are indeed the gold standard for establishing causality. However, as this manuscript focuses on a computational analysis of empirical transcriptomic and phenotypic datasets, our aim is to identify and quantify statistical associations and trends that are consistent with –and informative for– biological understanding, rather than to perform experimental validation.

Moreover, we would like to emphasize that our identification of ASK neurons in association with chemotaxis is not only statistically robust but also biologically meaningful. ASK neurons are well-established chemosensory neurons in *C. elegans*, with extensive literature supporting their involvement in the detection of environmental chemical cues and modulation of chemotactic behavior (e.g.,https://doi.org/10.1016/0896-6273(91)90276-6 and https://doi.org/10.1016/j.bbagen.2009.03.032). Thus, rather than proposing a novel or speculative link, our analysis recovers known biology providing a form of validation for our computational framework.

7. Independent, matched-resolution validation

The manuscript would benefit from independent validation using a dataset that aligns more closely in both temporal and spatial resolution with the embryonic single-cell transcriptomic data used in this study. While the authors rely entirely on the WPO, which is heterogeneous and coarse-grained, they cite, but do not incorporate, a more appropriate resource: the study by Xiao et al. (2022), which provides systematically measured embryonic phenotypes at single-cell resolution following gene perturbation. This dataset offers a rare opportunity to validate the D–P rule under more controlled and resolution-matched conditions. Using it to test whether genes with similar developmental expression profiles also yield similar embryonic phenotypes would directly support (or challenge) the study's central claim. At minimum, comparison of trends between the WPO-based analysis and those derived from the Xiao dataset would clarify the generalizability of the findings.

We now include a detailed discussion of the Xiao *et al.* dataset in the Supplement (section 'The D–P rule under tightly controlled, resolution-matched conditions') and have updated the manuscript accordingly. While we acknowledge its value, the dataset's limited scope makes it suboptimal for validating our organism-wide, integrative analysis. Our study spans 8,233 genes with

developmental expression and phenotypic annotations covering nearly 80% of phenotyped *C. elegans* genes and capturing a broad, organism-level phenotype space. In contrast, Xiao *et al*. examine 752 genes (restricted to chromosome I and human orthologs) and assess only six early phenotypes related to proliferation, differentiation, and morphogenesis, up to the 350-cell stage. Similarly, Green et al. (https://doi.org/10.1016/j.cell.2024.04.012), which we reference in the Supplement (section 'Alternative measures of pleiotropy'), covers 500 genes and early tissue-level features.

While high-resolution perturbation datasets offer important developmental insights, their limited gene and phenotype coverage make them insufficient for validating the genome-scale statistical relationships we explore. Our approach is designed to identify general principles linking developmental expression to diverse phenotypic outcomes, leveraging the most comprehensive annotation resources currently available.

8. Overstatement of conclusions

At times, the manuscript overstates the strength of its findings. The observed correlation between expression and phenotype similarity is only moderate and largely average-case. Interpretations of exceptions to the rule (e.g., D-p or d-P genes) remain speculative without empirical follow-up. The conclusions would benefit from a more cautious and nuanced tone.

We have revised the manuscript to adopt a more balanced tone.

We believe these revisions address the reviewer's concerns and improve the clarity and interpretive rigor of the manuscript. We appreciate the thoughtful feedback.

Reviewer #2:

Comments

First paragraph on Page 3: this is the most important paragraph of the Introduction. I think I follow the flow and logic, but a schematic showing the different questions and associated metrics would improve comprehensibility.

* I assume that Figure 1A is a version of what I am requesting for a new Introductory Figure (#1). If so, please use this as the basis for the new Figure 1.

We have followed the reviewer's advice and created a new figure that closely aligns with what the reviewer described. While this content builds upon the logic of Figure 1A, we chose to place the new schematic overview in the supplement (now Figure S1) for a deliberate reason: Figure 1A is meant to provide a *high-level conceptual overview* that eases readers into the narrative without overwhelming them with technical detail. Introducing too much complexity in the main text too early risks disrupting the flow and accessibility of the Introduction and early Results. By situating the more detailed schematic in the supplement, we preserve this readability while still making the logic and structure of our approach fully transparent to interested readers.

A few words about the D-P rule:

* this definition would probably only hold true for C. elegans.
* as a general phenomenon, you are essentially talking about developmental buffering. If would be nice to talk a bit more about the cell-specific mechanisms you propose for each version of D-P, (perhaps in the context of the lineage tree. I know that you mention the C. elegans lineage tree in passing, but this needs a more in-depth discussion.

We thank the reviewer for its comment and agree that the fully determined lineage of *C. elegans* is an important point that should be addressed carefully. Indeed, this organism, with its fully mapped and invariant cell lineage, represents a particularly useful model to investigate whether similar cell division history (i.e., lineages) lead to similar phenotypes.

However, this question falls somewhat outside the conceptual framework we propose with the D-P rule. Rather than relying on lineage information, our approach is centered on cell identity: we define gene expression during development using single-cell data, where each cell is annotated with an estimated embryonic time and a specific cell type or subtype. This embryonic/cell type definition of gene developmental expression, makes the D-P rule applicable beyond *C. elegans*, which would not be feasible if we depended solely on lineage-based information, as more complex organisms do not have fully mapped linages.

As shown in Supplementary Figure S6C–D (in new version Supplement) and the figure below (figure 1 for reviewer #2), although *C. elegans* has a well-defined and invariant lineage, many cells in the dataset lack lineage annotations, while cell type identities are more comprehensively resolved. This is particularly true at later developmental stages, when cells are already differentiated and can be readily identified using specific markers. However, without lineage tracking or prior

knowledge of the exact terminal identity, lineage cannot be inferred from markers alone. By organizing our developmental space around cell types and their temporal emergence, we address this limitation and provide a framework applicable to other species.

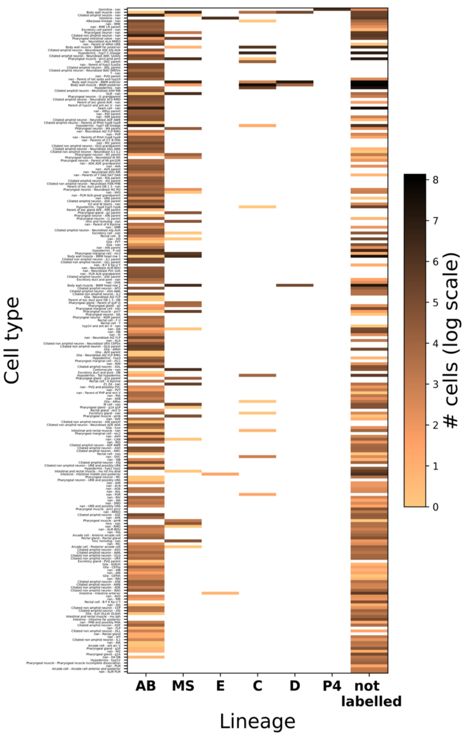

***Figure 1 for reviewer #2.** Link between cell type and lineage. For each single cell tagged with a specific cell type, we find its progenitor lineage (if it is annotated). We see that the major part of cells is unclassified or belong to AB lineage. Cells that belong to mc3 cell type are associated with two lineages with the following label: MSxaapapa/ABalpappapp.*

Following the reviewer's question about the broader applicability of our approach, we developed the same analysis that led to the D-P rule in zebrafish (*Danio rerio)* in a new supplementary section. Zebrafish shows a similar tendency to that of *C. elegans*, in an organism that exhibits different developmental dynamics. We expect this to provide additional evidence supporting the validity of our approach across more diverse biological systems.

Figures in Results:

Figure 2: interesting approach in using barcodes to provide intepretability to the different cases of convergence and divergence in development. Yet it might also help to annotate the figure a bit more so that the patters we are looking for are made explicit.

We have added further annotations to Figure 2 to make the relevant patterns and comparisons more explicit, as suggested. Additionally, we direct the reviewer to the Supplement (section 'Deviations of the D-P rule'), where we provide a detailed discussion of the differences in phenotypic profiles across the various cases.

* I am also not clear on how to interpret Figure 3. For the most part, this figure is difficult to interpret, but Figure 3C helps to summarize the significance of the analysis.

We thank the reviewer for the feedback. Note that Figs. 3A to 3C correspond to comparative analysis of the set of pleiotropic genes where developmental information is increasingly considered.

With respect to Fig. 3A, we analyzed expression breadth by calculating, for each gene, the fraction of sampled cells (independent of cell type or embryonic time) in which the gene is *active*. These values were then averaged across pleiotropic genes to generate a summary score, with the same approach applied to non-pleiotropic genes. Pleiotropic genes are expressed in significantly more cells than expected, whereas non-pleiotropic genes show limited expression and no enrichment for phenotypes or GO terms. The figure in question visualizes these two group-level scores alongside a *null* distribution generated by sampling random gene sets of comparable size, reinforcing the significance of the observed differences.

In Fig. 3B, we incorporated information about developmental time. This corresponds to two features that define the two axes of the figure: when is the first time that a given gene becomes expressed (`initial embryo time', x axis) and the range of time that a given gene is expressed (`# embryonic times', y axis). We mapped these two features for the set of pleiotropic (green dots) and non-pleiotropic (orange dots) genes. Finally, we show the distribution of the top 100 pleiotropic genes in the explicit (cell type, embryo time) space in Fig. 3C.

We have edited the main text to improve the understanding of the rationale of these figures.

* **Figure 4,** or the coarse interpretation of the D-P rule, is the least convincing part of this analysis. The "courseness" here refers to the cell types. It does seem like the deterministic cell fates of C. elegans will make these results a bit less generalizable.

We would like to highlight that the coarse interpretation of the D–P rule is supported by extensive analyses presented in the Supplementary Information, where we now demonstrate its applicability beyond *C. elegans* by generalizing the findings to zebrafish (see section in *new* supplement 'The D–P rule and its coarse interpretation in zebrafish'). This cross-species validation supports the broader relevance of the coarse-level interpretation, even in organisms with less deterministic developmental trajectories.

I like the "intrinsic features" section of the Discussion, which highlights the potential issues with generalizing from C. elegans development. **But you do need to speculate on how this D-P rule will vary or even be applicable across species.**

We appreciate the reviewer's positive feedback on the 'intrinsic features' section. In response to this suggestion, we have briefly expanded the Discussion to address how the D–P rule may be applicable across species. We also analyzed the rule in zebrafish as noted above. These additions aim to provide a broader perspective on the D–P rule.
