# Supplementary material for: Cell atlases and the developmental foundations of the phenotype
Source: PLoS Comput Biol. 2026 Feb 9;22(2):e1013944. doi: 10.1371/journal.pcbi.1013944 (PMC12904592; doi:10.1371/journal.pcbi.1013944)
Supplement: Attachment — Submitted filename: Lou_etal_revresponse.pdf [file pcbi.1013944.s014.pdf]

MINISTERIO
DE CIENCIA, INNOVACIÓN Y
UNIVERSIDADES

CSIC
CONSEJO SUPERIOR DE INVESTIGACIONES CIENTÍFICAS

Jan 20, 2026

This is a *minor* revision that required no explicit response to reviewers.

Sincerely,

Prof. Juan F. Poyatos

National Museum of Natural History, CSIC, Madrid.

juanfpoyatos@csic.es

National Museum of Natural Sciences (CSIC)
C/ José Gutiérrez Abascal, 2
28006 Madrid, Spain
Phone: +34 914111328 Ext. 443665
